# Diabetes care for people experiencing homelessness in the UK: insights from a national survey of frontline professionals and the development of an integrated care model

**DOI:** 10.3389/fpubh.2025.1672014

**Published:** 2025-10-13

**Authors:** Daniela Oehring, Martha Paisi, Mona Nasser, Theo Jackson, Ryan Young, Lynne Wooff, Helen Partridge, Jacqueline Conaty, Samantha Dorney-Smith

**Affiliations:** ^1^Faculty of Health, School of Health Professions, University of Plymouth, Plymouth, United Kingdom; ^2^Faculty of Health, Peninsula Dental School, University of Plymouth, Plymouth, United Kingdom; ^3^Pathway, London, United Kingdom; ^4^Brownlow Health General Practice, Liverpool, United Kingdom; ^5^Bolton Diabetes and Endocrine Service, Bolton Foundation Trust, Bolton, United Kingdom; ^6^Bournemouth Diabetes and Endocrine Service, University Hospitals Dorset, Bournemouth, United Kingdom

**Keywords:** ill-housed persons, diabetes mellitus, healthcare disparities, patient care management, health services accessibility

## Abstract

**Introduction:**

People experiencing homelessness (PEH) face food insecurity, unstable housing and fragmented services that render conventional diabetes pathways unworkable and amplify complications.

**Methods:**

Between January and April 2024, we conducted a nationwide, cross-sectional mixed-methods survey of front-line professionals via NHS, inclusion-health and voluntary-sector networks, analysing quantitative data (*n* = 104) with ANOVA, Kruskal–Wallis tests and ordinal logistic regression, and subjecting free-text responses to reflexive thematic analysis, before converging findings to develop the Integrated Holistic Diabetes Care Model for Homelessness (IHD-CMPH).

**Results:**

Respondents comprised specialist diabetes clinicians (31%), homelessness/inclusion-health staff (38%) and VCSE providers (32%); median perceived Type 1 prevalence among PEH was 20% versus 8% nationally (*p* < 0.001). Fifty-seven per cent rated diabetes outcomes for PEH as poor or very poor, and 66% reported more frequent amputations and vision loss. Clear organisational policies (OR 1.62, 95% CI 1.06–2.48), cross-sector collaboration (OR 2.76, 1.20–6.36) and outreach-specific training (OR 2.50, 1.50–4.17) were independently associated with better outcomes. Thematic analysis highlighted service fragmentation, inflexible appointments and insufficient homelessness-specific education.

**Discussion:**

Diabetes inequities among PEH stem chiefly from modifiable structural failures rather than patient non-adherence. The novel IHD-CMPH, anchored in outreach and mobile screening, provides a scalable framework to operationalise inclusion-health policy, improve glycaemic surveillance and avert avoidable admissions; this first national study translating multi-sector front-line evidence into a coherent policy model offers concrete levers for health-system reform and equity advancement.

## 1 Introduction

Homelessness and diabetes intersect to create serious public-health and clinical challenges. In the United Kingdom, people experiencing homelessness (PEH) encompass rough sleepers, sofa-surfers, people in temporary or unsafe housing (e.g., hostels, vehicles, survivors of domestic violence) (1–3). During 2023–24, 358,370 households in England underwent a statutory homelessness assessment, a 10.4% rise on the previous year; 178,560 were already homeless, and 146,430 were threatened with homelessness (4). By March 2024, 117,450 households were residing in temporary accommodation, representing a 12.3% year-over-year increase (5). These trends reflect rising rent arrears, tenancy loss and reduced asylum support.

Diabetes arises when the pancreas produces insufficient insulin or when insulin action is impaired. The two principal forms are Type 1 (6) and Type 2 (7). Type 3c diabetes, often alcohol-related, involves pancreatic damage and impaired insulin production and is relevant to PEH (8, 9).

For individuals with diabetes, homelessness imposes practical and psychosocial barriers: safe storage of insulin, regular meals, stigma, mobility between services and attendance at follow-up without a fixed address (10–15). Although prevalence studies are scant, existing evidence suggests that overall diabetes prevalence among PEH approximates that of the housed population (16–20), with an Irish estimate of about 8% (18). Nevertheless, PEH experience markedly higher rates of macrovascular disease, acute glycaemic emergencies and skin infections (2, 21), magnified by inconsistent healthcare access, poverty and unstable housing.

These challenges are intensified for vulnerable subgroups. Ethnic minority and immigrant PEH face added barriers, language discordance, mistrust, and service exclusion, disrupting diabetes management and care continuity (22). Sensory impairments also amplify risk: visual loss hinders glucose monitoring and medication use; hearing loss leads to communication breakdowns without adequate support (23, 24). However, data on these groups remains limited, underscoring the need for targeted outreach and accessible services.

Diabetes care for PEH is delivered by a wide network of professionals (25): (i) NHS Specialist Diabetes Services (SDS), (ii) NHS Homeless/Inclusion Health Services (HIS) providing outreach or street medicine, and (iii) NHS and Voluntary, Community and Social Enterprise (VCSE) providers who facilitate appointments, medicines and basic support. Fragmentation between these sectors means that many patients move through mainstream, specialist, and voluntary services without receiving cohesive care (26). International attempts to integrate outreach, telemedicine or hostel-based pharmacies into coordinated models repeatedly falter due to housing insecurity, food scarcity, policy constraints and underfunding (2, 10, 27, 28). Success depends on multi-sector collaboration, flexible scheduling and inter-professional coordination (10, 29).

Evidence points to promising components, context-specific education (28, 30), peer mentoring (31), digital tools linked to community outreach (32–34), and cross-disciplinary training (16, 35, 36), yet critical gaps persist in understanding how clinicians navigate structural and psychosocial complexities in unstable environments (10, 37, 38). Most interventions assume stable accommodation, refrigeration and continuous records, overlooking the tacit knowledge required when continuity and trust are disrupted.

Robust UK-wide evidence on how frontline professionals manage diabetes among PEH, which organisational factors aid success and how these insights can inform integrated policy, is lacking. To address this gap, we undertook a national mixed-methods survey within a 15-month quality improvement programme (39). Our objectives were to (i) document professionals’ perceptions of diabetes prevalence and complications in PEH, (ii) evaluate how reported challenges influence management outcomes, and (iii) identify organisational and service-level strategies that could enhance cross-sector collaboration, training and policy reform.

## 2 Materials and methods

This cross-sectional survey adheres to the STROBE guideline (40) for cross-sectional studies and incorporates key items from the CROSS checklist (41). Ethical approval was obtained from the University of Plymouth Faculty of Health Ethics Committee (#2023–4,667-5638) in accordance with the Declaration of Helsinki. Participants provided electronic informed consent via the JISC platform; no IP addresses or cookies were stored.

### 2.1 Survey development and piloting

A steering group of healthcare professionals and Experts by Lived and Living Experience of homelessness (Pathway, UK) guided study design, item generation, and piloting to ensure content validity. Twelve professionals and experts completed pilot testing (August–October 2023); their responses were excluded from analysis. The final instrument comprised 69 items across seven domains: (1) demographics and professional background; (2) perceived diabetes prevalence among PEH; (3) health outcomes; (4) screening and assessment; (5) training and preparedness; (6) accessibility, outreach and engagement; and (7) service improvement. Perceived prevalence items covered Type 1, Type 2, and Type 3c (pancreatogenic) diabetes, and the proportion requiring insulin therapy. Adaptive branching targeted relevant questions to specific provider groups (HIS, SDS, HCP; Table 1), reducing respondent burden (completion time approximately 25 min). To mitigate bias, questions were neutrally phrased, branching logic minimised survey fatigue, and responses were fully anonymous. The full questionnaire is available in Supplementary Table S1.

**Table 1:** Roles of different service providers in diabetes care for people experiencing homelessness (PEH) in the UK, and rationale for group-specific survey items.

Service type	Core role/functions	Examples of services provided	Why some survey items were targeted
Homeless/inclusion health services (HIS)	Specialist primary care and outreach for excluded groups, often multidisciplinary.	Street medicine, hostel/day centre clinics, outreach assessments, integration with housing/welfare.	Items on outreach, engagement, peer support, and opportunistic screening were targeted, reflecting their frontline role in mobile and inclusion health.
Specialist diabetes services (SDS)	Hospital- and community-based multidisciplinary diabetes teams delivering structured biomedical care.	HbA1c monitoring, retinopathy and foot screening, insulin initiation/titration, acute inpatient care.	Items on structured diabetes processes of care and biomedical management were directed at SDS respondents.
Other NHS and voluntary, community and social enterprise providers (HCP)	General practice, allied health, and VCSE organisations offering access, coordination, and support.	Appointment facilitation, medication supply, health promotion, addressing social determinants (e.g., food and housing).	Items on outreach and social support were targeted, given their emphasis on access pathways and addressing social determinants.

### 2.2 Recruitment and sample size

Purposive and snowball sampling were conducted between January and April 2024 through national inclusion health networks (e.g., Faculty for Homeless and Inclusion Health; Diabetes Specialist Nurse Forum UK). Eligible respondents worked in healthcare or VCSE services and routinely managed or supported diabetes care for PEH. A minimum of 96 respondents was required to achieve 95% confidence with a 10% margin of error for prevalence estimates and 0.3 precision for Likert-scale items.

#### 2.2.1 Data management

Survey data were exported to secure University of Plymouth OneDrive storage. Records with >30% missing data were excluded. Item-level non-response was <10%, and case-wise deletion was used to avoid the distributional assumptions of multiple imputation or maximum likelihood. Likert items were numerically coded.

#### 2.2.2 Statistical analysis

Analyses were performed in Python (Jupyter Lab-Desk V4.2.5–1), with *α* set at 0.05. Descriptive statistics included frequencies, means, medians, and standard deviations. Between-group differences on ordinal items were tested with the Kruskal–Wallis H test; significant results were explored with Mann–Whitney U tests and Bonferroni correction. Associations between categorical variables and group membership were assessed with Chi-squared tests. For normally distributed interval-scale data, one-way ANOVA was used.

Because perceived proportions of Type 1 and Type 2/3 diabetes are complementary, within-group dominance was tested using a one-sample Wilcoxon signed-rank test of deviation from 50%. Between-group comparisons of Type 1% used Kruskal–Wallis tests with Mann–Whitney post-hoc comparisons. Perceived prevalence was benchmarked against national estimates using one-sample Wilcoxon tests. Effect sizes were reported as η^2^ (Kruskal–Wallis: small ≥0.01, medium ≥0.06, large ≥0.14) and Cramér’s V (Chi-squared: small ≥0.1, medium ≥0.3, large ≥0.5). Pairwise Spearman correlation coefficients were calculated for outcomes and complications and visualised as a symmetric heatmap.

Ordinal logistic regression examined predictors of care quality, perceived difficulty, and preparedness. Significant univariate predictors were entered into multivariate models. Assumptions were tested: the proportional odds assumption (Brant test) showed no violations affecting interpretation, and variance inflation factors (VIF) were <5, indicating no problematic multicollinearity. Although predictor overlap attenuated some effect sizes, diagnostics confirmed no bias. Model fit was reported with McFadden’s pseudo-R^2^, and results as *β*-coefficients, odds ratios (OR), and 95% confidence intervals (CI).

#### 2.2.3 Qualitative analysis

Open-ended responses were analysed reflexively using Braun and Clarke’s six-phase thematic approach (42). Two researchers independently coded responses, resolving discrepancies by consensus. Themes were refined iteratively for coherence and distinctness (overview in Supplementary Table S2).

#### 2.2.4 Integration

Quantitative and qualitative findings were triangulated to contextualise statistical patterns and to inform the development of the Integrated Holistic Diabetes Care Model for People Experiencing Homelessness (IHD-CMPH).

## 3 Results

A total of 104 valid responses were analysed. An overview of the full results is provided in Supplementary Table S3. All participants practised in England: 32 (30.8%) worked in NHS Specialist Diabetes Services (SDS), 39 (37.5%) in NHS Homeless/Inclusion Health Services (HIS) and 33 (31.7%) in other NHS or VCSE provider roles (HCP). Most respondents (91%) were urban-based, with the highest concentrations in London (40%) and the North-West (18%). Regional distribution differed significantly across service groups (V = 0.389, *p* = 0.006); the West Midlands was the only region unrepresented.

Professionally, 42% were nurses and 22% medical doctors or general practitioners; the remainder comprised dietitians, allied health professionals and VCSE staff. HIS respondents mainly worked in primary-care or GP settings (44%) and community teams (31%); SDS respondents worked in community teams (50%) and acute hospitals (47%); HCP respondents mainly were in third-sector organisations (52%) or NHS services (24%). Respondents were experienced: 18.5 years in practice, 9.6 with PEH, 6.0 in current roles.

### 3.1 Perceptions of care quality, difficulty and preparedness

**Figure 1:**
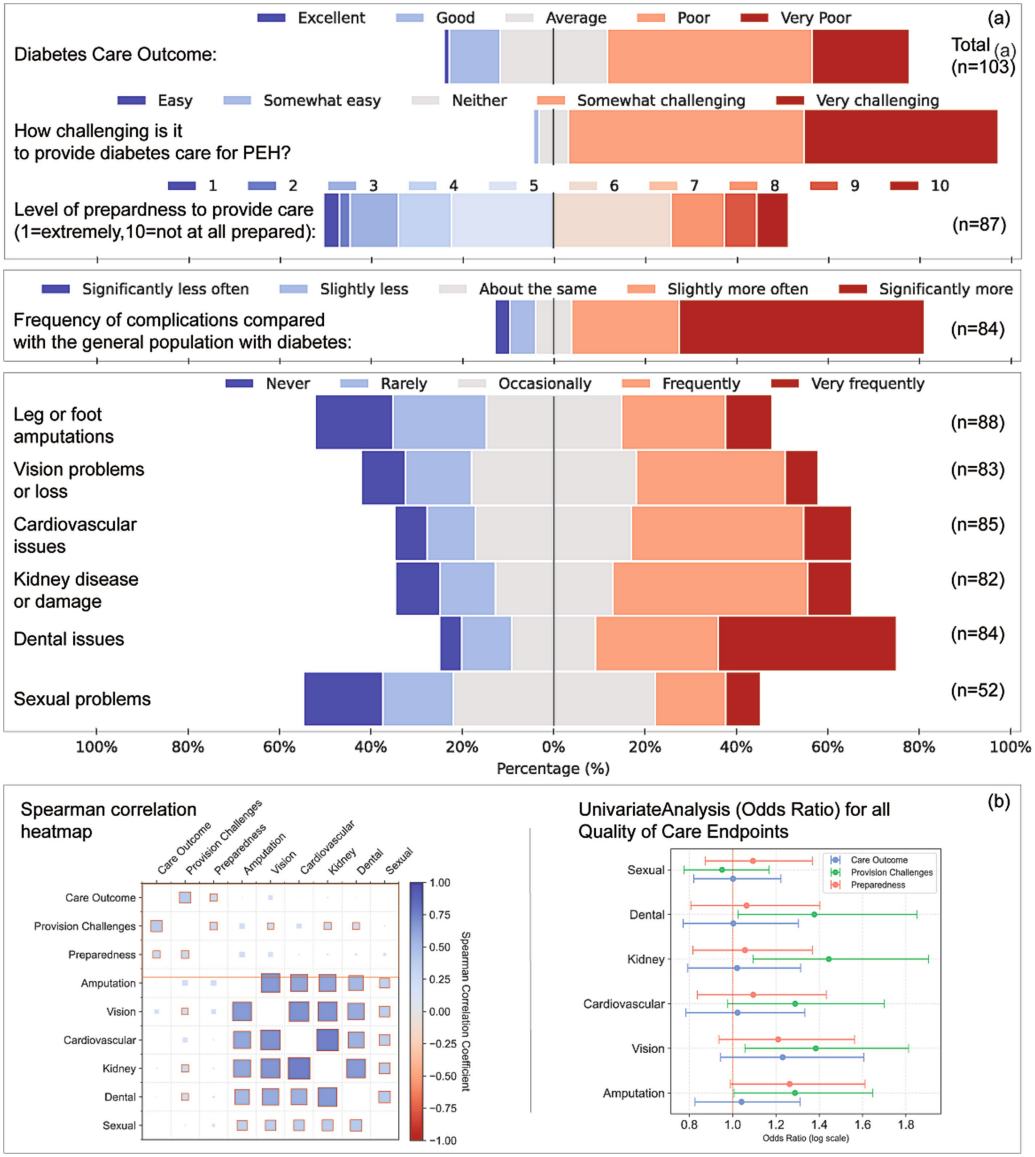
Perceptions of care quality, preparedness, complication frequency, and analytical associations for diabetes care among people experiencing homelessness (PEH). **(a)** Respondent ratings on five domains: Perceived overall diabetes care outcomes for PEH (*n* = 103), Level of challenge in providing care (*n* = 101), Preparedness to provide care, rated on a 10-point scale (*n* = 87), Perceived frequency of diabetes-related complications in PEH compared with the general diabetes population (*n* = 84), and Frequency of specific complications experienced by PEH (*n* = 52–88), including leg or foot amputations, vision loss, cardiovascular issues, kidney damage, dental problems, and sexual health concerns. **(b)** Left: Spearman correlation heatmap displaying relationships among perceived care outcomes, provision challenges, preparedness, and complication frequency. Both axes display the same variables, and each cell indicates the Spearman’s *ρ* correlation coefficient (colour intensity reflects strength and direction of the association). Right: Univariate odds ratio analysis for each complication type, estimating associations with care outcomes, perceived provision challenges, and preparedness levels. Odds ratios are plotted on a logarithmic scale; error bars represent 95% confidence intervals.

Three key metrics assessed perceptions of diabetes care for PEH: overall care outcomes, perceived difficulty in managing diabetes, and preparedness to provide care, (Figure 1). Fifty-seven per cent of respondents rated overall diabetes outcomes for PEH as poor or very poor (HIS 63%, SDS 66%, HCP 42%; *p* = 0.516). One HIS respondent noted, “*Outcomes are consistently poor*” (ID4), and an SDS participant described “*very challenging (…) conditions that aren’t improving*” (ID43). Poorer ratings were predicted by greater perceived difficulty (*β* = 0.696, OR = 2.01, *p* < 0.001) and lower preparedness (*β* = 0.405, OR = 1.50, *p* = 0.015).

One-third (33%) described diabetes management in PEH as somewhat or very challenging; this view did not differ by service type (*p* = 0.452) but was associated with higher difficulty scores (*β* = 0.717, OR = 2.05, *p* < 0.001) and lower preparedness (β = 0.355, OR = 1.43, *p* = 0.028). Overall, 49% felt adequately prepared (median 6/10). Higher preparedness predicted greater confidence with complex presentations (*β* = 0.229, OR = 1.26, *p* = 0.012).

### 3.2 Diabetes-related complications

Sixty-six per cent believed complications occurred more often in PEH than in the general diabetes population (Figure 1). Frequent leg or lower limb amputations were reported by 32% of SDS, 28% of HIS and 27% of HCP respondents (η^2^ = 0.047, *p* = 0.035). Vision loss was rated frequent by 34% of SDS and occasional by 36% of HIS and 31% of HCP (V = 0.317, *p* = 0.025). HIS perceived cardiovascular complications most often (41%). Dental problems were rated “*very frequent*” by 51% of HIS (η^2^ = 0.044, *p* = 0.041). Sexual-health complications were largely unreported (ns).

A third (32%) attributed care challenges to the instability of PEH lives, active substance use, mental illness, and transience. One HIS respondent explained, “*Patients are in active addiction and lead chaotic lifestyles… often do not have the motivation to engage with specialist services*” (HIS, ID1). 28% highlighted the mismatch between structured care and chaotic lifestyles. An SDS respondent noted, “*The care offered is good, but uptake is poor… appointments missed, follow-ups not made*” (SDS, ID30), and another added, “*They move and cannot access medication or appointments*” (HIS, ID94). 26% cited structural barriers, including inadequate housing support, language issues, and a lack of storage or transportation. “*No fridge for insulin storage… insulin stolen… no transport for appointments*” (SDS, ID53), and “*Literature not available in other languages*” (HCP, ID50). Respondents also reported frequent complications, including neuropathic pain (20%), foot infections (23%), and diabetic ketoacidosis (23%).

### 3.3 Perceived diabetes prevalence

**Figure 2:**
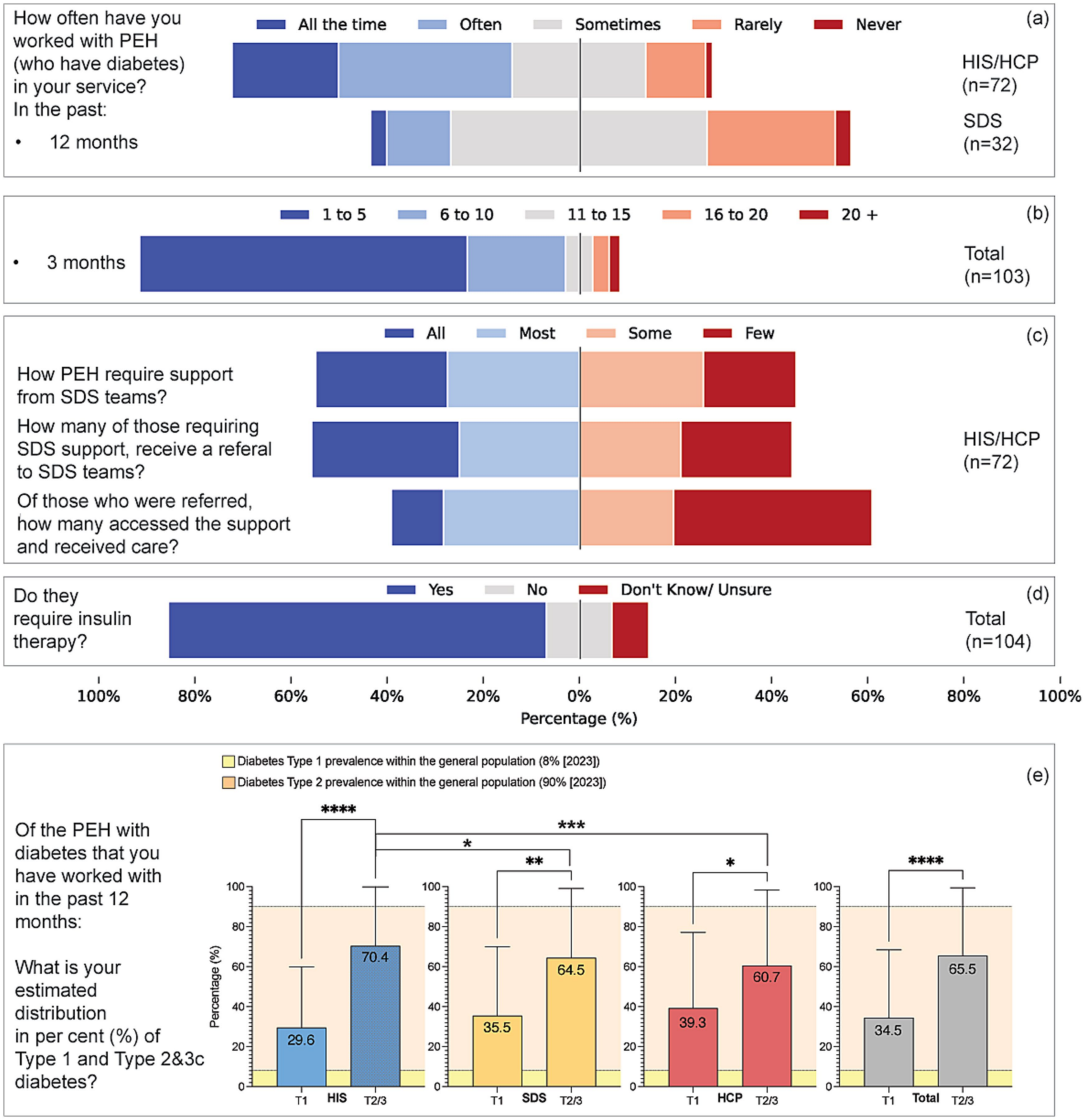
Patterns of service engagement, support pathways, insulin therapy, and perceived diabetes type distribution among people experiencing homelessness (PEH). **(a)** Reported frequency of working with PEH who have diabetes over the past 12 months, comparing HIS/HCP (*n* = 72) and SDS (*n* = 32) respondents. **(b)** Estimated number of PEH with diabetes seen in the past 3 months (*n* = 103). **(c)** Perceptions among HIS/HCP respondents (*n* = 72) regarding the extent to which PEH require support from SDS, are referred to SDS, and subsequently access and receive care. **(d)** Perceptions of whether PEH with diabetes therapy of insulin is needed among all respondents (*n* = 104). **(e)** The estimated prevalence of diabetes types among PEH in the past 12 months is shown as a mean percentage by respondent group (HIS, SDS, HCP, and total sample). Bars show perceived Type 1 and Type 2/3 proportions (mean ± SD) by provider group. Within-group inference tests whether Type 1% differs from 50% (one-sample Wilcoxon); between-group differences are tested on Type 1% (Kruskal–Wallis/Mann–Whitney). National benchmarks (Type 1 = 8%, Type 2 = 90%) are overlaid for context. Yellow-shaded areas represent Diabetes UK prevalence estimates for the general population in 2023 (Type 1: 8%; Type 2: 90%). Error bars indicate standard deviation. Asterisks denote levels of statistical significance: *p < 0.05, **p < 0.01, ***p < 0.001, ****p < 0.0001.

The mean perceived prevalence of Type 1 diabetes among PEH was 34.5% (median 20%, SD = 31.5; *n* = 86) (Figure 2). For combined Type 2/Type 3c diabetes, the mean was 65.5% (median 70%, SD = 35.6). The median perceived Type 1 prevalence exceeded the national population estimate of 8% (z ≈ 5.87, *p* < 0.001), whereas the perceived type 2/3c prevalence was lower than the population estimate of 90% (43) (*p* < 0.001). Seventy-nine per cent had managed PEH requiring insulin in the previous year, ranging from 90% in HIS and 88% in SDS to 56% in HCP (V = 0.276, *p* = 0.003).

When stratified by provider type, perceptions of prevalence and care varied. HIS respondents reported higher perceived prevalence of insulin dependence and more frequent barriers related to chaotic lifestyles, whereas SDS emphasised structured care processes but noted uptake challenges. HCP perceived a lower prevalence and reported less preparedness. These patterns indicate that provider experience and role context influence reported prevalence and care perceptions. By contrast, geographical setting showed little variation; 91% of respondents practised in urban areas, and regression models did not demonstrate significant associations between urban versus non-urban location and perceptions of prevalence or care outcomes.

### 3.4 Access to screening and support services

**Figure 3:**
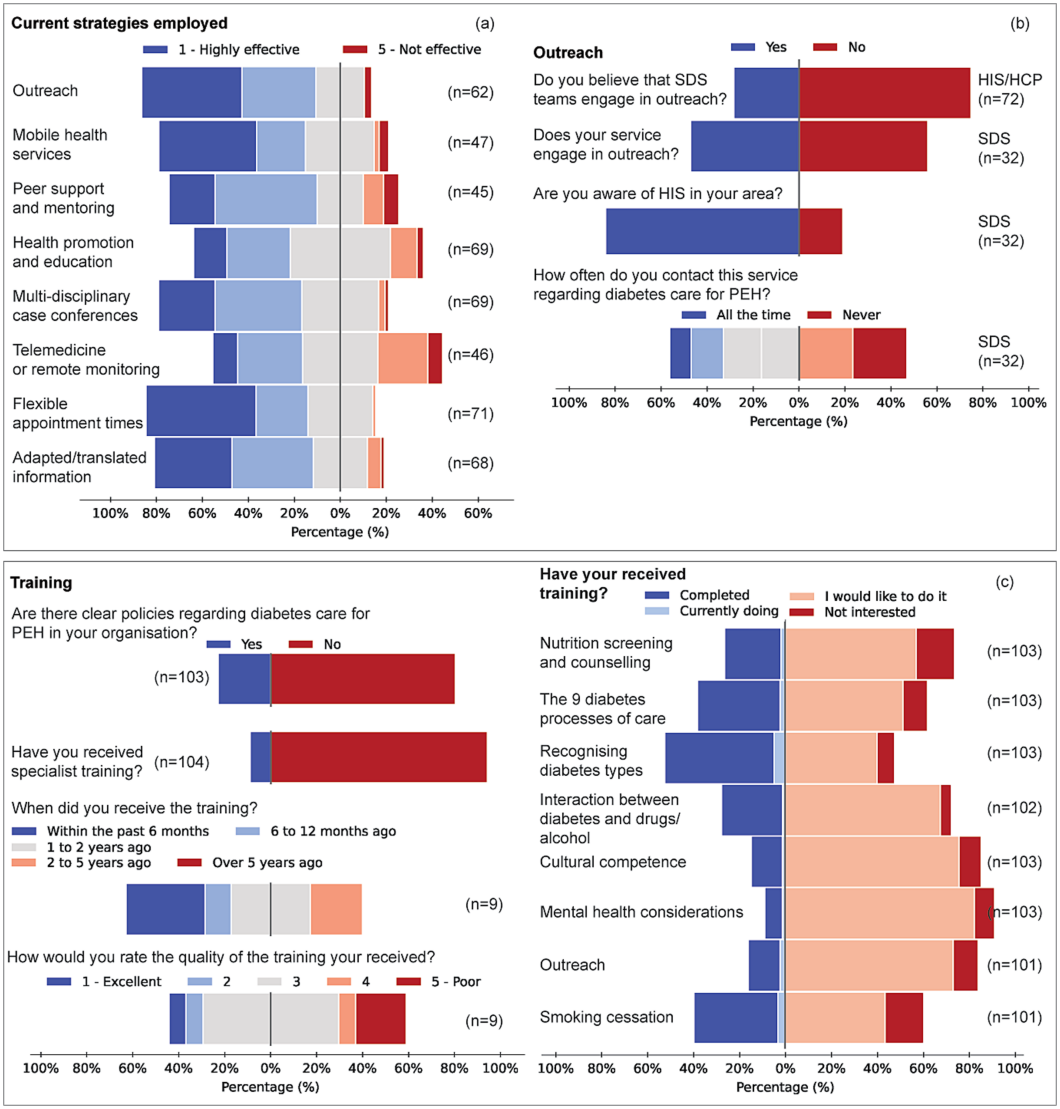
Structural processes, access barriers, and perceived ease of diabetes care delivery for people experiencing homelessness (PEH). **(a)** A proportion of respondents reported whether diabetes screening and housing status assessments are included in the standard assessment process for new referrals. Responses are disaggregated by service group (HIS/HCP, *n* = 72; SDS, *n* = 32). **(c)** Reported ease of access to diabetes screening tests (left) and support services (right). Screening includes Hba1c, fasting blood sugar (FBS), oral glucose tolerance test (OGTT), random blood sugar, blood pressure checks, foot and eye examinations, kidney function tests, and nutrition screening. Support services include smoking cessation, alcohol and drug misuse support, dietary input, mental health care, and exercise prescription. **(c)** Barriers hindering diabetes care for PEH, Items represent pooled responses from all service groups (HIS, SDS, HCP). Left: factors affecting PEH’s access to care (e.g., substance misuse, mistrust, poor diabetes awareness, financial issues). Right: barriers affecting healthcare professionals’ ability to provide care (e.g., insufficient training, limited resources, patient complexity, difficulty with follow-up, collaboration challenges).

HIS were more likely than other groups to embed diabetes screening in standard assessments (58% vs. 34% SDS and 18% HCP; V = 0.424, *p* = 0.002) (Figure 3). Over half of SDS services did not record housing status at referral, and 65% received no housing information. Access to HbA1c testing was rated “*very easy*” by 34% of HIS; 31% of SDS and 51% of HCP lacked this information (*p* < 0.001, η^2^ = 0.180). Similar patterns were seen for fasting/random glucose tests, foot and kidney assessments. Access to the oral glucose-tolerance test (OGTT) was reported as difficult across all groups.

HIS more often reported easy access to smoking-cessation support (35%) compared with considerable uncertainty among SDS and HCP (*V* = 0.437, *p* < 0.001). Dietetic input was difficult for 43% of HIS respondents, and mental-health services were rated “*very difficult*” by 30% of HIS (*V* = 0.379, *p* = 0.049). Barriers related to complex social needs were cited by 79% of HIS, 64% of SDS and 40% of HCP (*V* = 0.350, *p* = 0.002); patient mistrust was identified by 71% of HIS (*V* = 0.358, *p* = 0.001).

### 3.5 Management strategies and training

**Figure 4:**
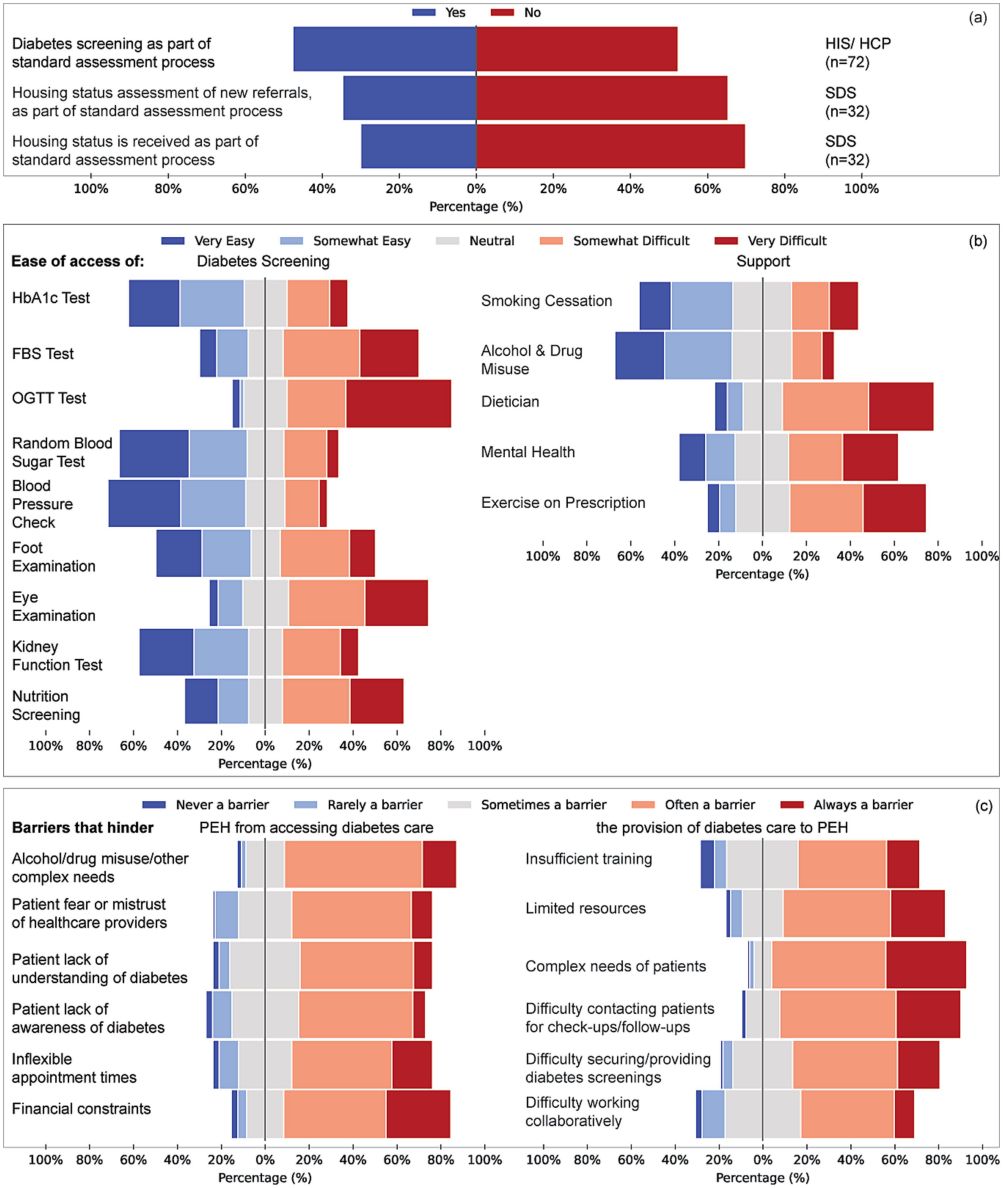
Engagement strategies, outreach activity and workforce training for diabetes care among PEH. **(a)** Perceived effectiveness of seven engagement strategies, outreach programmes, mobile-health services, peer support and mentoring, health promotion and education, multidisciplinary case conferences, flexible appointment times and adapted/translated information, rated on a five-point scale (1 = highly effective, 5 = not effective). **(b,c)** Items represent pooled responses from all service groups (HIS, SDS, HCP). **(b)** Service-level outreach activity: proportion of respondents whose service undertakes outreach (yes/no) and reported frequency of contact with local specialist homeless services (all the time, sometimes, never). **(c)** Training: percentages of respondents who have received homelessness-focused diabetes training (yes/no), the recency of that training (≤ 6 months to > 5 years) and its perceived quality (1 = excellent, 5 = poor). Training demand across eight topic areas (core diabetes processes, nutrition counselling, recognition of diabetes types 1/2/3c, interaction between diabetes and substance use, smoking-cessation support, cultural competence, mental-health comorbidity and outreach/access skills), showing proportions who have completed, are currently undertaking, would like, or are not interested in each topic.

Across all domains, respondents tended to feel confident in their speciality (e.g., diabetes management for SDS, homelessness/addiction for HIS) but reported limited competence in complementary domains, highlighting the need for coordinated, cross-disciplinary training (Figure 4).

Outreach was deemed highly effective by 39% of HIS, 13% of SDS and 24% of HCP; over half of SDS (53%) and 42% of HCP marked it “*not applicable*” (V = 0.243, *p* = 0.063). Peer support received “*very effective*” ratings from 39% of HIS but was rarely used by SDS (68% N/A) or HCP (54% N/A). Mobile-health approaches differed significantly between groups (η^2^ = 0.062, *p* = 0.017); HIS rated them more effective than SDS (*p* = 0.006) and HCP (*p* = 0.020). Flexible appointment times were favoured by 50% of HIS versus 21% of SDS (*V* = 0.312, *p* = 0.010). These differences reflect underlying practice models. HIS respondents, whose core remit includes outreach and street medicine, rated outreach as highly effective in improving access and outcomes. By contrast, outreach was not routinely embedded in SDS or HCP roles, which focus more on structured clinical services or third-sector support; over half of SDS and 42% of HCP respondents therefore marked outreach as “not applicable.” Importantly, the survey did not identify significant demographic differences in the PEH populations served across groups; the variation appears to reflect service scope rather than patient characteristics.

Tailored training was lacking for 87–96% of respondents. HIS respondents most often received training within the past 6 months; HCPs, 1–2 years ago. SDS had the longest gap, with only 3% trained in the past 2–5 years, and none within the past year. The quality of available training was generally rated as average (median 3/5). Half of all respondents reported no clear organisational policy on homelessness-related diabetes care.

Specific training topics revealed significant unmet needs. For nutrition screening and counselling, 65% of HIS, 46% of SDS, and 56% of HCP indicated they had not received training but would welcome it (*p* = 0.276). In contrast, SDS reported a 71% completion rate in training on the nine diabetes processes of care, compared to 30% for HIS and 6% for HCP (V = 0.411, *p* < 0.001). Recognition of Type 1/2/3c patterns showed a similar gradient (SDS 75%, HIS 43%, HCP 25%; V = 0.312, *p* = 0.003). Demand for additional training on substance use, smoking cessation and mental-health comorbidity was high across groups (Figure 4). Qualitative responses supported these findings and recommended blended, practice-focused training, as well as improved inter-service communication.

Respondents consistently reported confidence in their own speciality but limited competence in complementary domains. For example, SDS staff were confident in diabetes management but less so in addressing homelessness, addiction, or trauma; HIS respondents were skilled in outreach and psychosocial care but less confident in structured diabetes processes. Across groups, major unmet training needs included nutrition screening and counselling, substance-use management, mental health comorbidity, smoking cessation, and culturally adapted communication. These complementary domains were highlighted as essential to achieving diabetes care competence in homeless populations.

### 3.6 Ordinal logistic regression

#### 3.6.1 Perceived care outcomes

In univariate models six factors were associated with better overall outcomes for PEH: higher ratings for outreach (*β* = 0.23, OR = 1.26, 95%CI 1.06–1.49, *p* = 0.011), mobile-health services (*β* = 0.24, OR = 1.28, 1.07–1.54, *p* = 0.007), peer support and mentoring (β = 0.24, OR = 1.27, 1.05–1.53, *p* = 0.014), health promotion and education (*β* = 0.36, OR = 1.43, 1.15–1.78, *p* = 0.001), completion of specialised training (*β* = 0.95, OR = 2.60, 1.04–6.45, *p* = 0.040) and the presence of clear organisational policy (*β* = 0.48, OR = 1.62, 1.06–2.48, *p* = 0.027). When entered together, none retained significance (*β* range 0.04–0.56; all *p* > 0.18) and the model explained 5.8% of variance (McFadden’s R^2^ = 0.058), indicating considerable overlap between predictors.

#### 3.6.2 Perceived difficulty of diabetes management

Univariately, greater outreach effectiveness (*β* = 0.18, OR = 1.19, 1.00–1.42, *p* = 0.048) and more frequent service contact (*β* = 0.89, OR = 2.44, 1.23–4.87, *p* = 0.010) reduced perceived difficulty. In the multivariable model only contact frequency remained significant (*β* = 0.76, OR = 2.15, 1.06–4.35, *p* = 0.034); model fit McFadden’s R^2^ = 0.033.

#### 3.6.3 Preparedness to provide diabetes care

Univariate analyses showed higher preparedness among respondents who rated peer support favourably (*β* = 0.31, OR = 1.36, 1.12–1.65, *p* = 0.002), endorsed effective health promotion (*β* = 0.22, OR = 1.25, 1.02–1.54, *p* = 0.033), used adapted or translated information (*β* = 0.19, OR = 1.21, 1.00–1.45, *p* = 0.049) and had undertaken outreach-specific training (*β* = 0.86, OR = 2.36, 1.43–3.89, *p* < 0.001). Multivariable analysis retained peer support (*β* = 0.35, OR = 1.42, 1.10–1.84, *p* = 0.007) and outreach training (*β* = 0.92, OR = 2.50, 1.50–4.17, *p* < 0.001), explaining 6.6% of variance (McFadden’s R^2^ = 0.066).

#### 3.6.4 Summary of robust predictors

Across the three outcome domains, the variables that consistently retained independent associations were (i) frequent contact with PEH (linked to lower management difficulty), (ii) effective peer support and mentoring, and (iii) targeted outreach training (both linked to improved preparedness and perceived outcomes). These findings highlight the central role of regular engagement and cross-disciplinary skill-building in improving diabetes care for PEH.

### 3.7 Development of the integrated holistic diabetes care model for people experiencing homelessness (IHD-CMPH)

**Figure 5:**
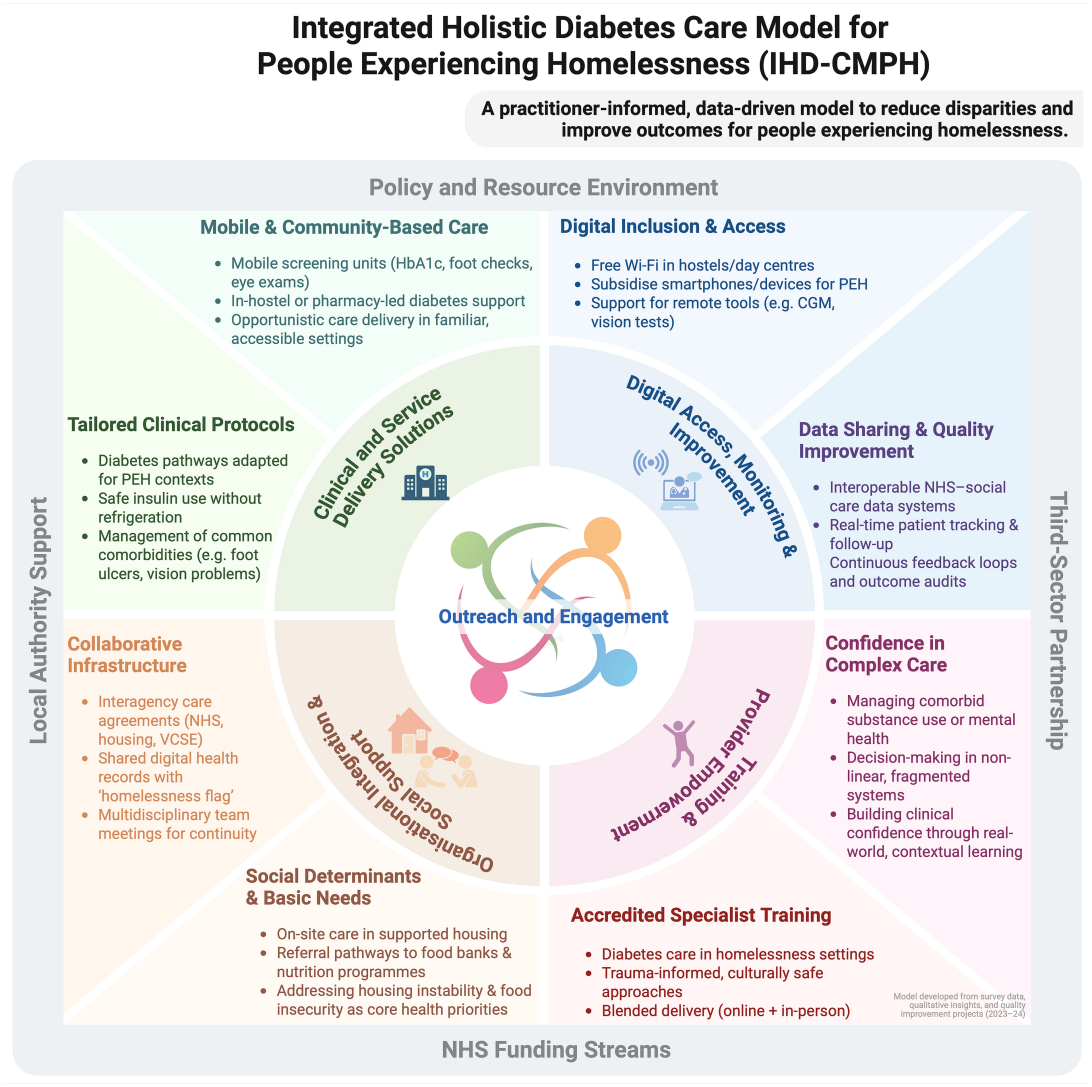
The Integrated Homelessness Diabetes Care Model for People Experiencing Homelessness (IHD-CMHP) diagram illustrates a comprehensive framework for addressing diabetes care in homeless populations. It encompasses multiple layers of support and intervention, structured around the core elements of clinical innovation, organisational efficiency, and social support. This model highlights the integration of Training, Digital Literacy, and Provider Empowerment within a supportive Policy and Resource Environment facilitated by Local Authority Support, NHS funding streams, and Third-Sector Partnerships. Key components include mobile health services, digital inclusion, Housing First initiatives, and multidisciplinary teams aiming to streamline care, enhance accessibility, and improve health outcomes. The diagram serves as a guide for implementing a holistic approach to healthcare, tailored to meet the unique challenges faced by people experiencing homelessness with diabetes, promoting sustainability and effectiveness in service delivery [Created in BioRender. Oehring, D. (2025), https://BioRender.com/q7i4o59].

Mixed-methods triangulation of (i) quantitative predictors, (ii) qualitative themes, and (iii) lessons from linked quality-improvement projects (39) informed a theory-driven framework for equitable diabetes care (Figure 5; Table 2). Multivariable analyses showed that frequent service contact independently reduced perceived management difficulty, while effective peer support and outreach-specific training independently increased preparedness and, in univariate models, perceived care quality. Qualitative data echoed these findings, with practitioners stressing that “*having services available within hostel settings (…) improves outcomes and access*” (HIS ID2). Participants also highlighted structural barriers, unstable accommodation, food insecurity and service fragmentation, and called for cohesive, interprofessional pathways.

**Table 2:** Evidence sources underpinning the integrated holistic diabetes care model for people experiencing homelessness (IHD-CMPH).

IHD-CMPH domain	Supported by survey analytics (quantitative predictors)	Supported by thematic analysis (qualitative data)	Supported primarily by literature/references
Clinical and service delivery solutions	Frequent service contact reduced perceived management difficulty; outreach effectiveness associated with better outcomes	Themes of fragmentation, inflexible appointments, and lack of access to screening	Reinforced by international evidence on outreach/mobile clinics
Organisational integration and social support	Clear organisational policy and cross-sector collaboration associated with better perceived outcomes (univariate)	Barriers included unstable housing, food insecurity, lack of coordination	Supported by NHS inclusion health frameworks and housing-linked care models
Training and provider empowerment	Outreach-specific training and peer support independently predicted preparedness and outcomes	Calls for blended, trauma-informed, practice-focused training	Supported by evidence on interdisciplinary and peer-mentor models
Digital access, monitoring and improvement	Not directly measured	Limited mention in qualitative responses (digital exclusion)	Strongly evidenced by literature on digital health equity and connectivity

Collectively, the evidence indicated that sustained, assertive outreach and cross-sector skill-sharing are foundational. The resulting IHD-CMPH (Figure 5) comprises four interrelated components, each anchored by an outreach ethos that delivers care within streets, hostels, day centres and other settings frequented by PEH:

1. *Clinical and service-delivery solutions* – assertive, community-based screening and follow-up (portable Hba1c, foot and retinal checks) plus in-hostel or day-centre clinics, underpinned by protocols addressing insulin storage, comorbid substance use and mental ill-health.
2. *Organisational integration and social support* – formal NHS–housing–VCSE agreements, a homelessness flag in electronic health records, and linkage to accommodation, food-bank and welfare services to mitigate social-determinant barriers.
3. *Training and provider empowerment* – accredited, trauma-informed modules delivered through blended learning to close the identified 87–96% training gap in homelessness-related diabetes care and to embed peer-mentor models.
4. *Digital access, monitoring and improvement* – Wi-Fi, subsidised devices, and interoperable data for remote monitoring and quality improvement.

While the IHD-CMPH is not a definitive solution, it integrates the statistically robust drivers (contact frequency, peer support, outreach training) with practitioner-defined needs, offering a coherent, evidence-informed template for scalable, person-centred diabetes care among PEH.

#### 3.7.1 Synthesis and model rationale

The IHD-CMPH translates the empirical signals from this study into a coherent, systems-level response; shifting toward an interdisciplinary, patient-centred approach tailored to the unique challenges of homelessness.

1. *Assertive, community-based clinical delivery*: Unstable accommodation and transience limit attendance at fixed-site services. The model therefore prioritises assertive outreach teams that deliver care in hostels, day centres and street settings and, where appropriate, employ mobile units equipped with point-of-care HbA1c analysers, foot-screening kits and handheld retinal cameras to provide opportunistic diagnostics and early intervention (44). In-hostel clinics and community-pharmacy partnerships extend this continuity, mirroring evidence that tailored outreach reduces emergency admissions through earlier detection and treatment.
2. *Organisational integration and social support*: Our data show that clear policies and adequate resources increase the odds of better perceived outcomes by 62%, and that intersectoral collaboration halves management difficulty (OR = 2.76 and 2.21 for key outreach variables). Accordingly, the IHD-CMPH calls for formal NHS–housing–VCSE agreements, routine multidisciplinary meetings, interoperable data-sharing systems and a homelessness flag in electronic health records to maintain continuity across highly mobile care trajectories (45). These mechanisms institutionalise joint accountability and minimise follow-up loss.
3. *Addressing social determinants and digital exclusion*: Housing instability, food insecurity and lack of connectivity surfaced repeatedly in qualitative accounts as barriers to glycaemic control. The model therefore embeds social-support pathways, links to Housing First schemes and local nutrition programmes, to secure the prerequisites for self-management (46, 47). Equally, subsidised devices and free Wi-Fi in accommodation enable engagement with telehealth and remote monitoring, reinforcing autonomy and continuity (48–52).
4. *Specialised workforce development*: With 87–96% of respondents lacking homelessness-specific diabetes training, the IHD-CMPH specifies accredited, trauma-informed curricula delivered through blended learning. Such programmes bridge the knowledge gap between diabetes, mental health and substance-use management and embed peer-mentor roles that our regression analyses associate with higher preparedness (30, 53–55).
5. *Economic rationale*: Integrated, outreach-oriented models consistently reduce emergency-department use and inpatient admissions, generating net savings within three to five years (14, 16, 56–58). Investment in assertive outreach infrastructure, interoperable data systems and joint commissioning is therefore fiscally prudent as well as clinically imperative (59–62).

By aligning clinical innovation with organisational integration, social-determinant mitigation and digital inclusion, the IHD-CMPH offers an evidence-based roadmap for equitable diabetes care in one of the UK’s most underserved populations.

## 4 Discussion

### 4.1 Summary of principal findings

This mixed-methods national survey demonstrates that diabetes care for PEH is hampered by a persistent design–reality gap: conventional appointment-centred pathways do not match the instability, multimorbidity and digital exclusion that characterise homelessness. Professionals perceived a markedly higher burden of type 1 and type 3c diabetes, frequent acute and chronic complications, and generally poor care outcomes. Quantitative analyses highlighted three modifiable levers - regular contact, peer support and outreach-specific training - while qualitative data underscored the need for integrated, flexible, cross-sector practice. These insights informed the IHD-CMPH.

### 4.2 Interpretation in relation to existing research

The perceived excess of amputations, retinopathy and cardiovascular disease aligns with earlier work linking housing instability to delayed diagnosis and treatment (46, 63). Similarly, respondents’ reports of dental problems corroborate evidence of high oral-health morbidity in homeless populations (11). Sexual-health complications were rarely recognised, mirroring wider diabetes services where fewer than one in ten patients are routinely asked about sexual concerns; this suggests an overlooked dimension of need (64). Our data also confirm that comorbid mental illness and substance use impede engagement (11, 28, 58, 65), reinforcing calls for opportunistic, outreach-based screening (44, 66, 67). Differences in perceptions between HIS, SDS and HCP respondents reflect role-specific exposures and service environments, consistent with qualitative accounts of fragmented pathways. Importantly, urban versus non-urban location did not materially affect reported prevalence or outcomes, likely reflecting the predominantly urban distribution of respondents.

Although epidemiological data specific to PEH are sparse, comparison with national audit benchmarks underscores a likely disparity. Chronic kidney disease affects around 30% of people with Type 2 diabetes in England (68), and major lower-limb amputation occurs at a rate of approximately 7.8 per 100,000 person-years (69). In contrast, our respondents reported much higher frequencies of these complications among PEH. While this may reflect genuine excess risk, as suggested by population-based cohort studies linking homelessness with higher complication rates (21), it may also be amplified by case-mix concentration in frontline services. This highlights the need for robust epidemiological studies quantifying complication burden among PEH.

At the organisational level, respondents working within services with clear homelessness policies and reliable resource access reported better outcomes, supporting studies linking structured pathways and staff support to continuity of care (45, 70). Equally, lower perceived management difficulty where strong health–housing collaboration existed echoes evidence for integrated, inclusion-health models (28).

The lower outreach ratings reported by SDS and HCP likely reflect role boundaries rather than differences in PEH demographics. HIS respondents routinely delivered street and hostel-based care, while SDS and HCP services operated primarily in structured or referral-based settings where outreach is less feasible or outside their mandate. This highlights the importance of cross-sector integration, whereby outreach capacity in HIS services is complemented by specialist input from SDS and support coordination from HCPs.

Collectively, these findings position homelessness as an upstream determinant of metabolic risk: insecure housing, food scarcity and limited connectivity directly undermine insulin storage, meal regularity and digital self-management (11, 29, 46). The IHD-CMPH addresses these realities by combining assertive community outreach, social-determinant mitigation and workforce development within an interoperable data architecture.

### 4.3 Strengths and limitations

Strengths include the first UK-wide sampling of front-line diabetes, inclusion-health and VCSE staff; a theory-informed instrument co-designed with Experts by Experience; and mixed-methods triangulation linking perceptions to explanatory themes.

This study’s cross-sectional design precludes causal inference; longitudinal evaluations are required to determine whether the strategies identified improve biomedical endpoints for PEH. Findings rely on professionals’ self-reports and may be affected by recall, reporting and social-desirability bias; absence of objective metrics limits external validity. Although the sample encompassed diverse NHS and VCSE settings, the numbers were modest (*n* = 104), and some regions, particularly rural areas and the West Midlands, were underrepresented, introducing potential selection bias. Service categories (SDS, HIS, HCP) facilitated analysis but may mask within-group heterogeneity.

The geographical distribution of respondents was skewed toward urban centres, particularly London, with under-representation of rural areas and no responses from the West Midlands. This imbalance may limit the generalisability of findings, as experiences of diabetes care for PEH in rural or under-sampled regions may differ, particularly in relation to service accessibility and integration. The sampling approach, while purposive and effective for engaging national inclusion-health networks, did not yield fully representative coverage. Future studies should seek broader regional participation to mitigate this limitation.

The explanatory power of the regression models was modest (pseudo-R^2^ 5–7%), indicating that the included predictors accounted for only a small proportion of variance in perceived outcomes. This is consistent with the complexity of diabetes care in PEH, where structural and contextual factors outside the scope of this survey, such as organisational culture, housing insecurity, inter-agency dynamics, and resource availability, likely play a greater role in shaping perceptions of quality, difficulty, and preparedness. Moreover, overlap between predictors reduced the independent effect of some variables in multivariate models, suggesting that provider training, organisational support, and outreach practices interact closely rather than exerting isolated effects. These limitations highlight the need for future research that integrates richer structural and contextual data to build more comprehensive predictive models.

Qualitative insights were derived from survey free-text responses rather than in-depth interviews, which restricted the exploration of tacit knowledge and inter-agency dynamics. This approach was selected to minimise respondent burden within a national survey and to capture perspectives from a broad and diverse sample. These constraints mean that unmeasured factors such as organisational culture or funding context could explain variance beyond that captured in our models.

### 4.4 Implications for practice and policy

The study suggests that incremental enhancements to standard diabetes pathways will be insufficient. Commissioners should: (i) embed assertive, multidisciplinary outreach clinics in hostels, day centres and primary-care networks; (ii) mandate homelessness flags in electronic records and formal health–housing collaboration agreements; (iii) fund accredited, trauma-informed training that bridges diabetes, mental health and substance-use expertise; (iv) invest in digital inclusion (Wi-Fi, devices) and interoperable data systems to support remote monitoring. Such reforms align with NHS Inclusion-Health ambitions and Core20PLUS5 equity goals, and international evidence indicates they are cost-saving within five years (47).

The findings suggest that competence in diabetes care for PEH requires integration of complementary domains beyond core diabetes knowledge. Specifically, training in trauma-informed care, substance-use and mental health management, culturally sensitive communication, and nutrition support were repeatedly identified as gaps. Embedding these domains within accredited, cross-sector curricula would help build the holistic competence needed for effective programme development.

### 4.5 Future research

Prospective implementation studies are needed to test the IHD-CMPH, measuring biomedical outcomes (HbA1c, admissions), costs and patient-reported experience. Realist and implementation-science designs will be valuable for unpacking context–mechanism interactions across urban and rural settings. Research should also examine the prevalence and management of type 3c diabetes and sexual-health problems, areas highlighted here but poorly documented.

We searched for comparative data from resource-rich UK centres in which provider perceptions of diabetes burden (complications, outcomes) are matched with objective frequencies. To our knowledge, no such study has been published in populations experiencing homelessness or similarly underserved groups. Existing UK studies, such as the observational urban vs. rural diabetes care study in England (71), which reports on care process metrics and treatment target achievement in general diabetic populations, offer useful benchmarks but do not directly measure provider perceptions. Similarly, national surveys such as the National Diabetes Experience Survey provide essential data on patient experience, but not matched objective complication burden per provider perceptions (72). The absence of such comparative studies underlines the importance of our findings. It indicates a gap in the literature: future research in well-resourced settings should combine provider perceptions with objective epidemiological data to determine the extent to which perception tracks genuine clinical need versus reflects resource limitations.

### 4.6 Conclusion

Poor diabetes outcomes among PEH reflect system failures, not clinician shortcomings. This study identified three actionable levers, regular contact, peer support, and outreach-specific training, and exposed how fragmented services, housing instability, and digital exclusion undermine care. The prevailing appointment-based model does not fit the realities of homelessness.

The Integrated Holistic Diabetes Care Model for Homelessness (IHD-CMPH) offers a practical, evidence-based solution. By embedding multidisciplinary outreach, tailored training, and data integration, it aligns with NHS inclusion-health policy and Core20PLUS5 goals. International data show such models reduce emergency admissions and save costs over time.

To drive change, future research must measure real-world outcomes and involve people with lived experience as co-designers. Closing the diabetes gap for PEH means redesigning care around mobility, instability, and trust. The path forward is clear; the challenge now is to take action.

## Data Availability

The original contributions presented in the study are included in the article/Supplementary material, further inquiries can be directed to the corresponding author/s.
