# Supplementary material for: Diabetes care for people experiencing homelessness in the UK: insights from a national survey of frontline professionals and the development of an integrated care model
Source: Front Public Health. 2025 Oct 13;13:1672014. doi: 10.3389/fpubh.2025.1672014 (PMC12554705; doi:10.3389/fpubh.2025.1672014)
Supplement: Supplementary file 1 [file Table_1.docx]

**Thematic Analysis ST1**

Q23: Of these patients requiring a referral but not receiving one, please describe why you think this is the case, if you feel able to do so.

Table 1. Q23 - Thematic analysis for the open-text responses. The table shows the overall theme, interpretation, and illustrative quote, as well as the number of participants. (HIS, HCP; n=37)

| **Theme** | **Interpretation** | **Illustrative Quote** | **HIS** | **HCP** |
| --- | --- | --- | --- | --- |
| **Service Accessibility Issues** | Challenges in accessing healthcare services due to systemic rigidity and patient lifestyle complexities. | "*Difficulty accessing or not engaging with the service we refer directly - despite support, clients don't attend due to rigidity of the service or alternatively due to chaotic lifestyle*" (ID 8) | 5 | 1 |
|  | High mobility and transient lifestyles of homeless populations hinder continuous care. | "*Transient nature of the patients*" (ID 14) | 0 | 1 |
| **Engagement and Compliance Challenges** | High rates of non-attendance at scheduled appointments due to unstable living conditions and other competing life challenges. | "*Patients have had a history of not attending appointments or struggling to engage with services so not referred by GP*" (ID 88) | 7 | 4 |
| **Systemic and Organisational Barriers** | Structural issues in healthcare service commissioning create barriers to accessing diabetes care. | "*The way services are commissioned in the city is a barrier*" (ID 7) | 1 | 1 |
|  | Lack of awareness among healthcare providers about specialised diabetes services, leading to underutilisation of available specialist care. | "*I didn’t know there was a specialised service- most of my diabetic clients receive treatment via their GP*" (ID 75) | 0 | 1 |
| **Patient-Centric Issues** | Competing health and life challenges often overshadow the management of diabetes, impacting the prioritisation of care. | "*Clients have co-occurring health problems and diabetes doesn't take priority*" (ID 13) | 0 | 2 |
|  | Patient autonomy influences care pathways; some patients refuse or are not eligible for specialist care. | "*I refer all patients that need it but only if they consent - some refuse onwards referral or are not eligible for secondary care input*" (ID 22) | 1 | 1 |
| **Communication and Information Gaps** | Lack of stable communication means due to homelessness complicates follow-ups and continuity of care. | "*People get lost in the system, have no postal address, can't receive letters, GP's aren't keeping on top of where the client is or how to contact them*" (ID 15) | 2 | 6 |
|  | Patients often lack necessary knowledge about their health needs and available services, which hinders effective management of diabetes. | "*Lack of knowledge and not being linked to primary care GP for regular monitoring*" (ID 94) | 3 | 1 |

Q25: Of these patients receiving a referral, but not accessing and receiving care, please describe why you think this is the case, if you feel able to do so.

Table 2. Q25 - Thematic analysis for the open-text responses. The Table shows the overall theme, interpretation, and illustrative quote, as well as the number of participants. (HIS and HCP; n=49)

| **Theme** | **Interpretation** | **Illustrative Quote** | **HIS** | **HCP** |
| --- | --- | --- | --- | --- |
| **Chaotic Lifestyles and Variable Engagement** | This theme reflects the unstable and unpredictable nature of homelessness, which significantly impacts individuals” ability to maintain consistent engagement with healthcare services. | “*Chaotic lifestyle making their engagement variable and often not engaging*” (ID 1) | 8 | 7 |
| **Service Inflexibility and Lack of Follow-Up** | Highlighting the need for healthcare systems to adapt to the unique challenges faced by homeless individuals, such as flexibility in appointment scheduling and active follow-up. | “*Non-flexible approach, discharged for non-attendance*” (ID 3) | 10 | 0 |
| **Logistical Barriers to Care** | These barriers encompass practical difficulties that prevent homeless individuals from accessing healthcare, such as communication issues and lack of a stable address for correspondence. | “*No fixed address, no mobiles, language barriers*” (ID 38) | 5 | 4 |
| **Competing Priorities** | Homeless individuals often prioritise immediate survival needs such as food and shelter over healthcare, illustrating a critical need for integrated support services. | “*Unable to attend appointments due to location, more pressing priorities*” (ID 13) | 2 | 4 |
| **Psychosocial Barriers** | Emotional and psychological barriers, including stigma and fear, deter engagement with healthcare services, underscoring the need for compassionate and understanding care approaches. | “*Fear of having to monitor their diet. Mental health barriers in attending appointments*” (ID 76) | 3 | 6 |

Q29: From the additional comments in this section (Q29, Free Text Answer, n=36), a clear pattern emerges around the multifaceted barriers to effective diabetes management within the homeless population, Table 3. Respondents highlighted critical challenges such as the lack of safe storage and access to insulin and supplies, as well as non-compliance and management difficulties due to chaotic lifestyles and limited education about diabetes care. Additionally, barriers to specialist care were evident, with frequent missed appointments and limited referral pathways alongside systemic healthcare barriers that restrict service provision. Finally, patient-centric barriers such as competing health problems and the need for responsive, flexible support further complicate care delivery. These insights underscore the complexity of providing diabetes care to this vulnerable population.

Table 3. Q29 - Thematic analysis for the open-text responses. The table shows the overall theme, interpretation, and illustrative quote, as well as the number of participants. (n=36)

| **Theme** | **Interpretation** | **Illustrative Quote with ID** | **HIS** | **SDS** | **HCP** |
| --- | --- | --- | --- | --- | --- |
| **Storage and Access Issues** | Difficulties in storing insulin and diabetes supplies, particularly for rough sleepers or those in unstable living conditions. | "*Nowhere safe to store insulin or things getting stolen and safe access to food are particular issues*" (ID 4) | 5 | 2 | 2 |
| **Non-Compliance and Management Challenges** | Struggles with adherence to diabetes management protocols due to lifestyle instability or lack of education about diabetes care. | "*due to rough sleeping and chaotic lifestyle and non-compliance, we have tried to support the client to have maximum oral therapy*..." (ID 2) | 6 | 1 | 3 |
| **Barriers to Specialist Care** | Obstacles in accessing specialised diabetes care, including lack of referrals or difficulties in reaching care facilities. | "*engagement is key. For the majority of the patients when referred they do not attend, miss appointments*..." (ID 12) | 2 | 2 | 4 |
| **Systemic Healthcare Barriers** | Structural issues within the healthcare system that prevent effective diabetes management for homeless populations. | "*The people I have worked with have temp accommodation and not rough sleepers*" (ID 30) | 0 | 2 | 2 |
| **Patient-Centric Barriers** | Challenges arising from the personal circumstances of patients, such as competing health problems or personal preferences affecting care access. | "*Responsive flexible support for those with insulin dependent diabetes that have a history of DKA/risk of death*..." (ID 35) | 1 | 1 | 3 |

Q30: Based on your professional experience, how would you rate diabetes care outcomes for individuals experiencing homelessness who have diabetes?

Table 4. Q31 - Thematic analysis for the open-text responses. The Table shows the overall theme, interpretation, illustrative quote, and the number of participants. (HIS, HCP; n=47 – 2 responses have been removed due to vagueness)

| **Theme** | **Interpretation** | **Illustrative Quote** | **HIS** | **SDS** | **HCP** |
| --- | --- | --- | --- | --- | --- |
| **Systemic Challenges vs. Individual Behaviour** | Respondents emphasise that homelessness-related chaos (addiction, mental health, transient lifestyles) directly undermines diabetes management, even when care is available. | *"Patients are in active addiction and lead chaotic lifestyles... often don't have the motivation to engage with specialist services"* (HIS, ID1).  *"Prioritising health can be very challenging with this group"* (HCP, ID52). | 9 | 0 | 6 |
| **Care Provision vs. Patient Access** | Tension between providers offering structured care (e.g., prescriptions, foot care) and patients” inability to engage due to homelessness-related barriers (e.g., no fixed address, competing priorities). | *"The care offered is good, but uptake is poor... appointments missed, follow-ups not made"*(SDS, ID30).  *"They move and cannot access medication or appointments"* (HIS, ID94). | 4 | 7 | 2 |
| **Structural Barriers to Care** | Systemic failures in housing, language support, and resource allocation (e.g., lack of fridges for insulin, inconsistent interpreting services). | *"No fridge for insulin storage... insulin stolen... no transport for appointments"* (SDS, ID53).  *"Literature not available in other languages"* (HCP, ID50). | 3 | 5 | 4 |
| **Provider Frustration and Burnout** | Respondents express frustration at perceived patient "non-compliance" despite efforts, highlighting emotional strain and systemic limitations. | *"Outcomes are poor due to lack of engagement despite our best efforts... it’s frustrating"* (SDS, ID41).  *"It’s easy to blame hospitals... reflects the hard lives of the homeless"* (HIS, ID102). | 1 | 1 | 1 |
| **Advocacy for Flexible, Trauma-Informed Care** | Calls for tailored approaches (e.g., outreach, trauma awareness) to bridge gaps between rigid systems and patient needs. | *"Assertive outreach works well... training hostel staff improves awareness"*(Group 1, ID85).  *"Outreach services need to exist to support clients"*(HCP, ID76). | 1 | 0 | 1 |

Q34: The responses (Q34, Free Text Answer to Q33.6 “Other”, n=56) indicate a complex interplay of direct medical complications related to diabetes and systemic issues affecting the homeless population. Neuropathic complications and wound-related problems are prevalent and are exacerbated by the living conditions associated with homelessness. Metabolic crises such as DKA and hypoglycaemia are particularly acute among those with inconsistent access to food and healthcare, reflecting the broader social determinants of health impacting this population. Infections and immune response issues are also significant, often related to poor nutritional status and environmental exposure. Access and systemic barriers are critical themes, especially for marginalised groups within the homeless population, such as pregnant women and those with substance use disorders. Mental health emerges as a critical component of overall health management, with many individuals struggling to cope with their diabetes diagnosis amidst the challenges of homelessness.

Table 5. Q34 - Thematic analysis for the open-text responses. The Table shows the overall theme, interpretation and illustrative quote as well as the number of participants (n=56).

| **Theme** | **Interpretation** | **Illustrative Quote** | **HIS** | **SDS** | **HCP** |
| --- | --- | --- | --- | --- | --- |
| **Neuropathic Complications** | Highlighting the prevalence of nerve damage and its impact on mobility and quality of life. | “*neuropathy, not leading to amputation but constant pain*” (ID 25) | 4 | 2 | 5 |
| **Wound-Related Issues** | Addressing complications related to unhealed wounds and infections, particularly foot ulcers in diabetic patients. | “*diabetic* *foot infections and ulcers. - they take up a significant amount of bed days in the patients we see in our service*” (ID 15) | 6 | 3 | 4 |
| **Metabolic Crises** | Focusing on acute management challenges such as diabetic ketoacidosis and hypoglycaemia. | “*DKA, hypos for those on insulin therapy - limited access to regular meals*” (ID 14) | 5 | 1 | 7 |
| **Access and Systemic Barriers** | Detailing challenges in accessing consistent healthcare and necessary medication due to systemic issues. | “*being able to* *administer insulin, due to being homeless and having nowhere safe to store medication, and or poor nutrition*.” (ID 12)  “*Struggle to access podiatry*” (ID 59) | 3 | 5 | 2 |
| **Infections and Poor Immune Response** | Discussing the higher risk of infections and poor immune response among homeless individuals. | “*Homeless people often present with diabetes harms* *DKA & HHS, low mood and depression, wound infections or slow healing wounds*.” (ID 74) | 2 | 1 | 2 |
| **Mental Health Complications** | Examining the mental health challenges and their direct impact on diabetes management. | “*Low* *mood. Exacerbated by diabetes*” (ID 88) | 1 | 0 | 3 |

Q41: Please describe any barriers you have experienced, with regards to securing/providing different diabetes screenings for people who are experiencing homelessness.

Table 6. Q41 - Thematic analysis for the open-text responses. The Table shows the overall theme, interpretation and illustrative quote as well as the number of participants. (n=63, HCP, SDS, HIS)

| **Theme** | **Interpretation** | **Illustrative Quote** | **HIS** | **SDS** | **HCP** |
| --- | --- | --- | --- | --- | --- |
| **Access and Engagement Barriers** | Challenges related to engaging patients in screening due to their lifestyles and systemic barriers within healthcare. | "*It can be challenging within our service to engage patients in screening that is not carried out by our service*." (ID 1) | 11 | 5 | 6 |
| **Systemic Healthcare Constraints** | Systemic issues within healthcare that prevent effective screening, such as service availability and bureaucratic hurdles. | "*Diabetes and inclusion health services differ in what they are able to offer on outreach (postcode lottery*)." (ID 38) | 3 | 5 | 3 |
| **Logistical and Infrastructure Challenges** | Physical and infrastructural barriers to accessing necessary screenings, such as transport and facility issues. | "*Transport issues are common e.g., for eye screening*." (ID 4) | 3 | 6 | 1 |
| **Resource Limitations** | Lack of resources within healthcare settings to properly screen or follow up with homeless individuals. | "*As a community team, we do not do bloods so are dependent on patient attending GP*." (ID 14) | 3 | 3 | 5 |
| **Social and Behavioural Factors** | Individual factors such as reluctance to engage, mental health issues, or lack of awareness that affect screening uptake. | "*Language barriers*." (ID 36) | 3 | 1 | 5 |

Q43: The open-text responses (Q43, Free Text Answer, n=32) reveal insights into healthcare providers' challenges in delivering diabetes care to homeless populations. Four key themes emerged, Table 7. Respondents frequently highlighted access and engagement barriers, with logistical and social factors limiting the continuity of care even when initial access to services was available. Social and structural barriers, such as regional disparities in service availability (e.g., exercise on prescription), further exacerbate inequities. Additionally, resource limitations, particularly regarding dietitian support, were noted as a significant gap in diabetes management for homeless individuals. The inadequate provision of mental health support and the broader need for integrated, continuous care were emphasised, pointing to systemic weaknesses in addressing the complex needs of this population.

Table 7. Q43: Thematic analysis for the open-text responses. The Table shows the overall theme, interpretation, and illustrative quote, as well as the number of participants (n=32).

| **Theme** | **Interpretation** | **Illustrative Quote** | **HIS** | **SDS** | **HCP** |
| --- | --- | --- | --- | --- | --- |
| **Access and Engagement Barriers** | Challenges in accessing services due to logistical, financial, or social barriers. | "*Accessing the teams that provide this type of health support is not difficult in the hospital but we find that following the initial assessment, there is little that can be done for our patients by these services*." (15) | 5 | 3 | 5 |
| **Social and Structural Barriers** | Barriers related to societal and structural issues that prevent effective care. | "*Exercise on prescription should be a no-brainer really, but this does seem to be a postcode lottery as well, and people often don't think to offer it to our patients*." (3) | 4 | 3 | 1 |
| **Resource Limitations** | Lack of resources and support services necessary to effectively manage health needs. | "*We work closely with all services outlined above except dieticians. However, this may be something we can improve on in the near future*." (2) | 6 | 1 | 0 |
| **Mental Health and Comprehensive Care** | Addressing mental health and the need for comprehensive, continuous care. | "*Mental health support is inadequate. I would be laughed at if I referred someone to the mental health team for support with diabetes*." (88) | 3 | 0 | 1 |

Q46: Please describe any other barriers that people experiencing homelessness face when engaging with their diabetes care.

Table 8. Q46: Thematic analysis for the open-text responses. The Table shows the overall theme, interpretation and illustrative quote as well as the number of participants (n=35, HIS, SDS and HCP).

| **Theme** | **Interpretation** | **Illustrative Quote** | **HIS** | **SDS** | **HCP** |
| --- | --- | --- | --- | --- | --- |
| **Access and Mobility Issues** | The lack of a fixed address, GP, or transportation significantly limits access to ongoing care and medication. | "*The main barriers hindering homeless people are not having a fixed address or GP. With an address or GP accessing support services including follow-up appts and obtaining repeat medications is extremely challenging if not impossible*. (ID 27)" | 1 | 1 | 0 |
| **Communication and Logistical Barriers** | Difficulties in communication and logistics significantly impede healthcare access and appointment adherence. | "*Accessing prescribed medications (insulins), sensors.* *Accessing diabetes education. Receiving letters/appointment information in a timely way.* (ID 46)" | 2 | 4 | 0 |
| **Comprehensive Health Barriers** | A broad range of health barriers, including mental health, that require integrated approaches to address effectively. | "*No cooking facilities, poor diet, moving from place to place, no fridge (for insulin) no trust in healthcare professionals, language barriers* (ID 33)" | 1 | 0 | 1 |
| **Cultural and Language Barriers** | Language and cultural differences create barriers in understanding and navigating healthcare services. | "*Language barriers, we have interpreters but not all areas do* (ID 25);" | 2 | 0 | 3 |
| **Logistical Barriers** | Geographical, timing, and facility-related challenges make it difficult to access care. | "*Stigma Locations Clinic appointments and timing* (ID 40)" | 0 | 1 | 0 |
| **Nutritional Challenges** | The high cost of healthy foods and lack of access to kitchen impacts what they can eat and when - healthy food is not available when they need to eat. | "*Healthy food is expensive and lack of access to kitchen impacts what they can eat and when - healthy food is not available when they need to eat*. (ID 18)" | 0 | 0 | 1 |
| **Psychological and Social Barriers** | Mental and social issues, including trauma and existential crises, impact motivation and care engagement. | "*Complex trauma and other life experiences past and present sometimes sadly mean patients can be in a state of not caring if they live or die. When in this state will be precontemplative of managing diabetes. Patients need a purpose, something to live and care for and to have hope.* (ID 35)" | 2 | 1 | 0 |
| **Reduced Barriers through Direct Care** | Barriers are minimised through direct and trust-based service delivery. | "*We bring most of the care to the clients so we perhaps have less barriers than other services. Clients know and trust us and will come to us for help* (ID 2)" | 1 | 0 | 0 |
| **Resource Limitations** | Limited availability of necessary medical supplies, medications, and educational resources restricts effective diabetes care. | "*Getting a hold of medication & supplies* (ID 74)" | 1 | 1 | 0 |
| **Societal and Professional Bias** | Prejudice and judgment from healthcare providers hinder access to care. | "*Prejudice from health care professionals.* (ID 4)" | 1 | 2 | 1 |
| **Storage and Access Issues** | Challenges in medication storage and accessing necessary facilities limit effective diabetes management. | "*No where to store insulin. Not attending GP surgery. (11); 'having a fridge to store their insulin, or glp-1's being able to eat any type of meal*. (ID 12)" | 2 | 0 | 3 |
| **Systemic Administrative Challenges** | Discharges and missed appointments due to system inefficiencies affect care continuity. | "*DNAs cause people being discharged (and people were often not aware of the appointments)* (ID 3)" | 1 | 1 | 0 |

Q54: Please describe any other strategies you use to improve accessibility and engagement for people experiencing homelessness with diabetes and/or any other strategies you feel are effective at improving service accessibility.

Table 9. Q54 - Thematic analysis for the open-text responses. The Table shows the overall theme, interpretation and illustrative quote as well as the number of participants. (n=23, HIS, SDS and HCP)

| **Theme** | **Interpretation** | **Illustrative Quote** | **HIS** | **SDS** | **HCP** |
| --- | --- | --- | --- | --- | --- |
| **Innovative Service Delivery Models** | Implementation of flexible and non-traditional healthcare models. | "*Education days for all homelessness services annually with speakers to raise awareness on all topics relevant to people experiencing homelessness*." (ID 85) | 4 | 0 | 1 |
| **Access and Engagement Challenges** | Barriers that prevent or make it difficult for homeless individuals to engage with health services. | "*Feel it is the means to executing diabetes more than the knowledge/education e.g. finances, cooking facilities/gym membership.*  *MDT discussions with health, housing and social work help to a degree but are still limited by social work and housing rules on limited housing stock/limited financial flexibility. Food vouchers are often unsuitable due to long-life/canned nature and halal patients report they are unsuitable for their needs. Some local fresh food initiatives would be great but need to take into account cooking facilities often kettle and shared microwave. A … initiative for those living under homelessness circumstances would be great taking into account how cooked/cost of electricity. This also instils recipe ideas for when move out of homelessness. "."* (ID 35) | 1 | 0 | 1 |
| **Systemic and Structural Barriers** | Systemic issues that hinder access to care and resources for homeless individuals. | "*Working in an acute Trust I have seen none of these strategies.*.." (ID 27) | 2 | 2 | 3 |
| **Resource and Infrastructure Limitations** | Challenges due to lack of necessary resources and infrastructure to support diabetes care. | " *There is very little out there that supports us in having strategies, aka we don't have access or know where to find.*" (ID 49) | 2 | 3 | 1 |
| **Cultural and Communication Barriers** | Difficulties arising from cultural differences and communication issues. | " *telephone interpreting service can be very unpredictable - no interpreters dropped calls*." (ID 94) | 1 | 0 | 2 |
