# Supplementary material for: Diabetes care for people experiencing homelessness in the UK: insights from a national survey of frontline professionals and the development of an integrated care model
Source: Front Public Health. 2025 Oct 13;13:1672014. doi: 10.3389/fpubh.2025.1672014 (PMC12554705; doi:10.3389/fpubh.2025.1672014)
Supplement: Supplementary file 2 [file Table_2.docx]

**Ordinal Regression Analysis ST2**

**Table ST2:** Overview of survey responses from healthcare professionals regarding care outcomes and challenges in managing diabetes among people experiencing homelessness (n=104). The table includes the most frequent answer (count/ sample size) of HIS, SDS, and HCP, along with P-values for chi-squared (χ²) and Kruskal-Wallis (KW) tests as well as the most frequent answers for the total cohort (count/ sample size). The effect of each question on the quality-of-care outcomes (overall care outcome, perceived difficulty in managing diabetes and preparedness to provide care) was evaluated using univariate ordinal logistic regression analysis. The effect is estimated using the regression coefficient β* and odds ratio (OR)**, and it is indicated if this is a significant contribution.

| **Question** | **HIS** | **SDS** | **HCP** | **P (χ^2^)**  **(Cramer V)** | **P (KW)**  **(η^2^)** | **Total** | **Overall care outcomes** | **Perceived difficulty in managing diabetes** | **Preparedness to provide care** |
| --- | --- | --- | --- | --- | --- | --- | --- | --- | --- |
| Please select the region where you primarily work: | London (18/37) | Southeast England (9/32) | London (19/33) | 0.006 (0.390) | 0.491 (-0.006) | London (41/102) | Beta=0.072, OR=1.07, p=0.314 | Beta=-0.036, OR=0.96, p=0.615 | Beta=0.067, OR=1.07, p=0.367 |
| Please indicate if your service is located in a rural or urban area. | Urban (36/39) | Urban (29/32) | Urban (30/33) | 0.963 | 0.964 (-0.019) | Urban (95/104) | Beta=-0.956, OR=0.38, p=0.126 | Beta=-0.267, OR=0.77, p=0.675 | Beta=-0.175, OR=0.84, p=0.785 |
| In the past 12 months, how often have you worked with people experiencing homelessness who have diabetes at your service? | Often (16/39) |  | Often (10/33) | 0.138 | 0.019 (0.064) | Often (26/72) | Beta=0.914, OR=2.49, p=0.000 | Beta=0.500, OR=1.65, p=0.023 | Beta=0.711, OR=2.04, p=0.002 |
| In the past 12 months, how often have you worked with people experiencing homelessness at your service? |  | Sometimes (16/32) |  |  |  |  | Beta=0.495, OR=1.64, p=0.120 | Beta=0.734, OR=2.08, p=0.030 | Beta=-0.019, OR=0.98, p=0.951 |
| In the past 3 months, how many people experiencing homelessness with diabetes have you worked with? | 1 to 5 (18/39) | 1 to 5 (18/32) | 1 to 5 (24/33) | 0.106 | 0.010 (0.071) | 1 to 5 (60/104) | Beta=-0.087, OR=0.92, p=0.469 | Beta=-0.265, OR=0.77, p=0.056 | Beta=-0.091, OR=0.91, p=0.499 |
| In your work with individuals experiencing homelessness who have diabetes, how many do you estimate require support from a specialist diabetes team? | Most (14/39) |  | All (10/32) | 0.045 (0.399) | 0.464 (-0.007) | All (17/71) | Beta=0.087, OR=1.09, p=0.579 | Beta=-0.170, OR=0.84, p=0.283 | Beta=0.083, OR=1.09, p=0.621 |
| Of those patients requiring support from a specialist diabetes team, how many do you think received a referral to such a team? | All (10/39) |  | I don't have this information. (9/33) | 0.065 | 0.036 (0.049) | All (16/72) | Beta=0.419, OR=1.52, p=0.001 | Beta=-0.071, OR=0.93, p=0.583 | Beta=0.299, OR=1.35, p=0.022 |
| Of those who were referred to specialist diabetes teams, how many do you think accessed the service and received care? | Few (12/39) |  | I don't have this information. (13/33) | 0.361 | 0.067 (0.034) | Few (19/72) | Beta=0.515, OR=1.67, p=0.001 | Beta=-0.071, OR=0.93, p=0.609 | Beta=0.497, OR=1.64, p=0.001 |
| During the past 12 months, have you worked with people experiencing homelessness who required insulin therapy? | Yes (35/39) | Yes (28/32) | Yes (18/32) | 0.003 (0.276) | 0.001 (0.124) | Yes (81/103) | Beta=0.656, OR=1.93, p=0.066 | Beta=0.599, OR=1.82, p=0.061 | Beta=0.437, OR=1.55, p=0.211 |
| **Based on your professional experience, how would you rate diabetes care outcomes for individuals experiencing homelessness who have diabetes?** | Poor (18/38) | Poor (12/32) | Poor (10/33) | 0.516 | 0.574 (-0.009) | Poor (40/103) | **Overall care outcomes** | Beta=0.696, OR=2.01, p=0.000 | Beta=0.405, OR=1.50, p=0.015 |
| Based on your professional experience, how frequently do you encounter diabetes-related complications amongst those experiencing homelessness with diabetes, compared to those in the general population with diabetes? | Significantly more (21/39) | Significantly more (18/32) | Slightly more often (9/33) | 0.422 | 0.227 (0.010) | Significantly more (48/104) | Beta=0.359, OR=1.43, p=0.028 | Beta=0.374, OR=1.45, p=0.043 | Beta=0.021, OR=1.02, p=0.900 |
| Based on your professional experience, how frequently do you encounter the following issues amongst patients who are experiencing homelessness with diabetes? (Please rate on a scale from 'Very often' to 'Never', using the provided matrix) | | | | | | | | | |
| - Leg or foot amputations | Occasionally (11/39) | Frequently (10/31) | Occasionally (9/33) | 0.044 (0.301) | 0.035 (0.047) | Occasionally (26/103) | Beta=0.040, OR=1.04, p=0.733 | Beta=0.253, OR=1.29, p=0.044 | Beta=0.234, OR=1.26, p=0.060 |
| - Vision problems or loss | Occasionally (14/38) | Frequently (11/32) | Occasionally (10/32) | 0.025 (0.317) | 0.102 (0.026) | Occasionally (30/102) | Beta=0.208, OR=1.23, p=0.125 | Beta=0.325, OR=1.38, p=0.018 | Beta=0.191, OR=1.21, p=0.143 |
| - Cardiovascular issues (e.g., heart disease, stroke) | Frequently (16/39) | Frequently (11/32) | Occasionally (11/33) | 0.174 | 0.479 (-0.005) | Frequently (32/104) | Beta=0.022, OR=1.02, p=0.870 | Beta=0.254, OR=1.29, p=0.073 | Beta=0.091, OR=1.09, p=0.508 |
| - Kidney disease or damage | Frequently (14/38) | Frequently (12/31) | Frequently (9/33) | 0.065 | 0.093 (0.028) | Frequently (35/102) | Beta=0.020, OR=1.02, p=0.875 | Beta=0.367, OR=1.44, p=0.009 | Beta=0.055, OR=1.06, p=0.674 |
| - Dental issues (e.g., abscesses) | Very frequently (20/39) | I don't have this information. (10/32) | Frequently (9/33) | 0.032 (0.308) | 0.041 (0.044) | Very frequently (32/104) | Beta=0.003, OR=1.00, p=0.981 | Beta=0.320, OR=1.38, p=0.034 | Beta=0.062, OR=1.06, p=0.658 |
| - Sexual problems (e.g., impotence) | I don't have this information. (14/39) | I don't have this information. (15/30) | I don't have this information. (21/33) | 0.341 | 0.136 (0.020) | I don't have this information. (50/102) | Beta=0.002, OR=1.00, p=0.987 | Beta=-0.050, OR=0.95, p=0.632 | Beta=0.090, OR=1.09, p=0.433 |
| Do your patients experiencing homelessness get screened for diabetes as part of your standard assessment process? | Yes (23/39) |  | No (19/33) | 0.002 (0.424) | 0.000 (0.163) | No (32/72) | Beta=0.384, OR=1.47, p=0.226 | Beta=-0.059, OR=0.94, p=0.850 | Beta=0.813, OR=2.25, p=0.017 |
| Do you assess the housing status of new referrals, as part of your standard assessment process? |  | No (17/32) |  |  |  |  | Beta=0.169, OR=1.18, p=0.660 | Beta=0.072, OR=1.07, p=0.852 | Beta=0.326, OR=1.39, p=0.510 |
| Do you receive information regarding the housing status of newly referred patients, as part of your standard referral process? |  | No (21/32) |  |  |  |  | Beta=0.476, OR=1.61, p=0.407 | Beta=-0.126, OR=0.88, p=0.821 | Beta=0.077, OR=1.08, p=0.889 |
| For each of the following types of diabetes screening, please indicate how easy it is for patients experiencing homelessness with diabetes to access them within the diabetes care process. (Please rate on a scale from "Very Easy" to "Very Difficult" for each screening type, matrix) | | | | | | | | | |
| - Haemoglobin A1c (HbA1c) Test: | Very Easy (13/38) | I don't have this information. (10/32) | I don't have this information. (17/33) | 0.003 (0.358) | 0.000 (0.180) | I don't have this information. (31/103) | Beta=0.182, OR=1.20, p=0.073 | Beta=0.041, OR=1.04, p=0.678 | Beta=0.288, OR=1.33, p=0.006 |
| - Fasting Blood Sugar (FBS) Test | Somewhat Difficult (12/38) | I don't have this information. (13/30) | I don't have this information. (19/33) | 0.040 (0.307) | 0.010 (0.073) | I don't have this information. (38/101) | Beta=0.150, OR=1.16, p=0.218 | Beta=0.108, OR=1.11, p=0.362 | Beta=0.244, OR=1.28, p=0.052 |
| - Oral Glucose Tolerance Test (OGTT) | Very Difficult (17/38) | I don't have this information. (18/31) | I don't have this information. (19/33) | 0.063 | 0.063 (0.036) | I don't have this information. (46/102) | Beta=0.306, OR=1.36, p=0.051 | Beta=0.422, OR=1.52, p=0.008 | Beta=0.193, OR=1.21, p=0.246 |
| - Random Blood Sugar Test | Very Easy (16/39) | I don't have this information. (12/31) | I don't have this information. (16/33) | 0.000 (0.409) | 0.000 (0.222) | I don't have this information. (31/103) | Beta=0.285, OR=1.33, p=0.004 | Beta=0.193, OR=1.21, p=0.044 | Beta=0.283, OR=1.33, p=0.007 |
| - Blood Pressure Check | Very Easy (17/39) | I don't have this information. (10/32) | I don't have this information. (14/33) | 0.000 (0.405) | 0.000 (0.228) | Very Easy (26/104) | Beta=0.272, OR=1.31, p=0.007 | Beta=0.184, OR=1.20, p=0.056 | Beta=0.328, OR=1.39, p=0.002 |
| - Foot Examination | Very Easy (11/39) | I don't have this information. (11/32) | I don't have this information. (14/33) | 0.005 (0.348) | 0.000 (0.151) | I don't have this information. (28/104) | Beta=0.311, OR=1.36, p=0.004 | Beta=0.255, OR=1.29, p=0.016 | Beta=0.412, OR=1.51, p=0.000 |
| - Eye Examination | Somewhat Difficult (11/39) | I don't have this information. (14/31) | I don't have this information. (14/33) | 0.010 (0.336) | 0.001 (0.126) | I don't have this information. (31/103) | Beta=0.456, OR=1.58, p=0.002 | Beta=0.506, OR=1.66, p=0.000 | Beta=0.415, OR=1.52, p=0.004 |
| - Kidney Function Test | Very Easy (13/39) | I don't have this information. (11/32) | I don't have this information. (17/33) | 0.003 (0.361) | 0.000 (0.150) | I don't have this information. (32/104) | Beta=0.245, OR=1.28, p=0.015 | Beta=0.090, OR=1.09, p=0.359 | Beta=0.266, OR=1.31, p=0.011 |
| - Nutrition screening using a recommended tool | Somewhat Difficult (8/39) | I don't have this information. (14/31) | I don't have this information. (18/33) | 0.031 (0.310) | 0.002 (0.103) | I don't have this information. (38/103) | Beta=0.229, OR=1.26, p=0.038 | Beta=0.193, OR=1.21, p=0.080 | Beta=0.325, OR=1.38, p=0.006 |
| For patients experiencing homelessness with diabetes, please indicate how easy it was for them to access the following types of health support (for those it is relevant to, for example cessation for smokers, alcohol team referrals for those with alcohol issues). | | | | | | | | | |
| - Smoking Cessation Support | Easy (14/39) | Don't Know/Unsure (18/32) | Neither Easy nor Difficult (9/33) | 0.000 (0.437) | 0.000 (0.238) | Don't Know/Unsure (29/104) | Beta=0.286, OR=1.33, p=0.008 | Beta=0.293, OR=1.34, p=0.007 | Beta=0.375, OR=1.46, p=0.001 |
| - Alcohol & Drug Misuse Support | Neither Easy nor Difficult (12/39) | Don't Know/Unsure (12/31) | Easy (11/33) | 0.003 (0.357) | 0.000 (0.156) | Easy (27/103) | Beta=0.450, OR=1.57, p=0.000 | Beta=0.391, OR=1.48, p=0.001 | Beta=0.329, OR=1.39, p=0.008 |
| - Dietician Support | Difficult (17/39) | Don't Know/Unsure (11/32) | Difficult (9/33) | 0.049 (0.297) | 0.434 (-0.003) | Difficult (33/104) | Beta=0.466, OR=1.59, p=0.001 | Beta=0.265, OR=1.30, p=0.061 | Beta=0.266, OR=1.30, p=0.065 |
| - Mental Health Support | Very Difficult (12/39) | Don't Know/Unsure (12/32) | Neither Easy nor Difficult (9/33) | 0.001 (0.379) | 0.003 (0.096) | Very Difficult (23/104) | Beta=0.483, OR=1.62, p=0.000 | Beta=0.473, OR=1.61, p=0.000 | Beta=0.302, OR=1.35, p=0.022 |
| - Exercise on Prescription | Difficult (13/39) | Don't Know/Unsure (15/31) | Don't Know/Unsure (12/33) | 0.047 (0.300) | 0.004 (0.090) | Don't Know/Unsure (34/103) | Beta=0.126, OR=1.13, p=0.359 | Beta=0.241, OR=1.27, p=0.085 | Beta=0.352, OR=1.42, p=0.014 |
| **Based on your professional experience, how difficult have you found managing diabetes for your patients who are experiencing homelessness / getting diabetes managed for your clients if you are a support worker?** | Somewhat challenging (16/36) | Very challenging (11/32) | Somewhat challenging (14/33) | 0.452 | 0.046 (0.042) | Somewhat challenging (40/101) | Beta=0.717, OR=2.05, p=0.000 | **Perceived difficulty in managing diabetes** | Beta=0.355, OR=1.43, p=0.028 |
| In your experience, how have the following barriers hindered patients experiencing homelessness from accessing diabetes care? | | | | | | | | | |
| - Alcohol/drug misuse/other complex needs | Often a barrier (31/39) | Often a barrier (20/31) | Often a barrier (13/32) | 0.002 (0.350) | 0.047 (0.042) | Often a barrier (64/102) | Beta=-0.237, OR=0.79, p=0.361 | Beta=-0.071, OR=0.93, p=0.790 | Beta=0.158, OR=1.17, p=0.570 |
| - Patient fear or mistrust of healthcare providers | Often a barrier (28/39) | Sometimes a barrier (14/31) | Often a barrier (14/33) | 0.001 (0.358) | 0.071 (0.033) | Often a barrier (56/103) | Beta=-0.094, OR=0.91, p=0.689 | Beta=-0.312, OR=0.73, p=0.159 | Beta=-0.185, OR=0.83, p=0.446 |
| - Patient lack of understanding of diabetes | Often a barrier (26/39) | Sometimes a barrier (14/31) | Often a barrier (13/33) | 0.026 (0.291) | 0.220 (0.010) | Often a barrier (53/103) | Beta=0.236, OR=1.27, p=0.290 | Beta=0.426, OR=1.53, p=0.077 | Beta=-0.035, OR=0.97, p=0.894 |
| - Patient lack of awareness of diabetes | Often a barrier (24/38) | Often a barrier (14/31) | Often a barrier (15/33) | 0.098 | 0.557 (-0.008) | Often a barrier (53/102) | Beta=0.099, OR=1.10, p=0.656 | Beta=0.341, OR=1.41, p=0.162 | Beta=-0.250, OR=0.78, p=0.317 |
| - Inflexible appointment times | Often a barrier (20/39) | Sometimes a barrier (13/31) | Often a barrier (14/33) | 0.156 | 0.325 (0.002) | Often a barrier (47/103) | Beta=0.292, OR=1.34, p=0.145 | Beta=0.083, OR=1.09, p=0.670 | Beta=1.000, OR=2.72, p=0.000 |
| - Financial constraints (e.g. being unable to pay for transport) | Often a barrier (17/38) | Often a barrier (17/31) | Often a barrier (13/32) | 0.044 (0.281) | 0.261 (0.007) | Often a barrier (47/101) | Beta=-0.251, OR=0.78, p=0.262 | Beta=-0.036, OR=0.96, p=0.866 | Beta=0.816, OR=2.26, p=0.001 |
| In your experience, how often have the following factors acted as barriers to providing quality diabetes care for patients experiencing homelessness? | | | | | | | | | |
| - Insufficient training around providing diabetes care/support for people experiencing homelessness | Sometimes a barrier (16/39) | Sometimes a barrier (10/31) | Often a barrier (13/33) | 0.099 | 0.639 (-0.011) | Often a barrier (37/103) | Beta=0.507, OR=1.66, p=0.002 | Beta=0.032, OR=1.03, p=0.834 | Beta=0.399, OR=1.49, p=0.020 |
| - Limited resources (e.g., funding, supplies, etc.) | Often a barrier (22/39) | Often a barrier (12/31) | Often a barrier (11/33) | 0.422 | 0.359 (0.000) | Often a barrier (45/103) | Beta=0.282, OR=1.33, p=0.137 | Beta=-0.071, OR=0.93, p=0.677 | Beta=0.664, OR=1.94, p=0.000 |
| - Complex needs of patients (e.g. substance misuse, mental health) | Often a barrier (23/39) | Often a barrier (17/31) | Often a barrier (12/33) | 0.218 | 0.864 (-0.017) | Often a barrier (52/103) | Beta=0.158, OR=1.17, p=0.534 | Beta=0.384, OR=1.47, p=0.150 | Beta=0.447, OR=1.56, p=0.100 |
| - Difficulty contacting patients for check-ups/follow-ups | Often a barrier (24/39) | Often a barrier (14/31) | Often a barrier (14/33) | 0.330 | 0.464 (-0.005) | Often a barrier (52/103) | Beta=0.309, OR=1.36, p=0.160 | Beta=0.272, OR=1.31, p=0.239 | Beta=0.602, OR=1.83, p=0.009 |
| - Difficulty securing/providing diabetes screenings for patients | Often a barrier (17/39) | Often a barrier (13/31) | Often a barrier (12/33) | 0.259 | 0.083 (0.030) | Often a barrier (42/103) | Beta=0.272, OR=1.31, p=0.117 | Beta=0.045, OR=1.05, p=0.797 | Beta=0.531, OR=1.70, p=0.005 |
| - Difficulty working collaboratively with other relevant services | Sometimes a barrier (16/39) | Often a barrier (12/31) | Often a barrier (14/33) | 0.711 | 0.534 (-0.007) | Often a barrier (41/103) | Beta=0.302, OR=1.35, p=0.098 | Beta=0.184, OR=1.20, p=0.284 | Beta=0.369, OR=1.45, p=0.040 |
| Please rate the extent to which the following improvements/ support would help you to overcome challenges in providing diabetes care to people experiencing homelessness (1 Very helpful - 5 Not helpful at all) | | | | | | | | | |
| - Additional resources for services (funding, staff, equipment etc.) | 1 - Very helpful (27/39) | 1 - Very helpful (18/32) | 1 - Very helpful (18/32) | 0.630 | 0.299 (0.004) | 1 - Very helpful (63/103) | Beta=0.503, OR=1.65, p=0.002 | Beta=0.299, OR=1.35, p=0.044 | Beta=0.003, OR=1.00, p=0.984 |
| - Training/education for healthcare staff on providing diabetes care for PEH | 1 - Very helpful (27/39) | 1 - Very helpful (14/32) | 1 - Very helpful (18/31) | 0.252 | 0.070 (0.034) | 1 - Very helpful (59/102) | Beta=0.184, OR=1.20, p=0.261 | Beta=0.193, OR=1.21, p=0.228 | Beta=-0.013, OR=0.99, p=0.936 |
| - Improved accessibility for healthcare appointments (e.g. flexible times) | 1 - Very helpful (31/39) | 1 - Very helpful (14/32) | 1 - Very helpful (22/33) | 0.007 (0.317) | 0.016 (0.062) | 1 - Very helpful (67/104) | Beta=0.013, OR=1.01, p=0.933 | Beta=0.250, OR=1.28, p=0.095 | Beta=-0.132, OR=0.88, p=0.380 |
| - Enhanced collaboration between healthcare and community organisations | 1 - Very helpful (33/39) | 1 - Very helpful (19/32) | 1 - Very helpful (25/32) | 0.208 | 0.042 (0.043) | 1 - Very helpful (77/103) | Beta=0.287, OR=1.33, p=0.167 | Beta=0.428, OR=1.53, p=0.028 | Beta=-0.197, OR=0.82, p=0.338 |
| - Enhanced collaboration between specialist and mainstream healthcare organisations | 1 - Very helpful (32/37) | 1 - Very helpful (17/32) | 1 - Very helpful (22/33) | 0.097 | 0.023 (0.056) | 1 - Very helpful (71/102) | Beta=0.222, OR=1.25, p=0.200 | Beta=0.231, OR=1.26, p=0.155 | Beta=-0.070, OR=0.93, p=0.677 |
| - Greater availability of educational materials on diabetes for patients | 1 - Very helpful (22/38) | 2 (12/32) | 1 - Very helpful (16/32) | 0.085 | 0.006 (0.082) | 1 - Very helpful (44/102) | Beta=0.132, OR=1.14, p=0.362 | Beta=0.417, OR=1.52, p=0.005 | Beta=-0.117, OR=0.89, p=0.415 |
| - Improved data sharing between relevant organisations | 1 - Very helpful (27/39) | 1 - Very helpful (18/32) | 1 - Very helpful (17/33) | 0.265 | 0.199 (0.012) | 1 - Very helpful (62/104) | Beta=0.169, OR=1.18, p=0.275 | Beta=0.213, OR=1.24, p=0.116 | Beta=-0.153, OR=0.86, p=0.249 |
| Which strategies do you currently use to engage patients experiencing homelessness in their diabetes care? Please select all that apply and rate the effectiveness of each strategy using the scale provided | | | | | | | | | |
| - Outreach programs | 1 - Highly effective (15/38) | N/A (18/32) | N/A (13/33) | 0.063 | 0.009 (0.075) | Code 6 (41/103) | Beta=0.230, OR=1.26, p=0.008 | Beta=0.177, OR=1.19, p=0.044 | Beta=0.181, OR=1.20, p=0.043 |
| - Mobile health services | N/A (17/37) | N/A (23/32) | N/A (15/33) | 0.144 | 0.195 (0.013) | Code 6 (55/102) | Beta=0.244, OR=1.28, p=0.007 | Beta=0.063, OR=1.07, p=0.464 | Beta=0.067, OR=1.07, p=0.448 |
| - Peer support and mentoring | N/A (17/37) | N/A (22/32) | N/A (18/33) | 0.179 | 0.141 (0.019) | Code 6 (57/102) | Beta=0.235, OR=1.26, p=0.013 | Beta=0.156, OR=1.17, p=0.098 | Beta=0.305, OR=1.36, p=0.002 |
| - Health promotion and education | 3 (12/37) | N/A (17/32) | 3 (11/33) | 0.043 (0.303) | 0.003 (0.099) | Code 6 (33/102) | Beta=0.357, OR=1.43, p=0.001 | Beta=0.172, OR=1.19, p=0.097 | Beta=0.223, OR=1.25, p=0.035 |
| - Multi-disciplinary case conferences and care coordination | 2 (11/37) | N/A (13/32) | N/A (14/33) | 0.341 | 0.034 (0.048) | Code 6 (33/102) | Beta=0.136, OR=1.15, p=0.152 | Beta=0.108, OR=1.11, p=0.263 | Beta=0.171, OR=1.19, p=0.079 |
| - Telemedicine or remote monitoring | N/A (21/36) | N/A (13/32) | N/A (20/32) | 0.193 | 0.053 (0.040) | Code 6 (54/100) | Beta=0.009, OR=1.01, p=0.934 | Beta=-0.046, OR=0.95, p=0.659 | Beta=0.026, OR=1.03, p=0.822 |
| - Flexible appointment times | 1 - Highly effective (19/38) | N/A (16/32) | 1 - Highly effective (12/33) | 0.010 (0.312) | 0.000 (0.138) | 1 - Highly effective (34/103) | Beta=0.055, OR=1.06, p=0.525 | Beta=0.054, OR=1.06, p=0.537 | Beta=0.161, OR=1.17, p=0.080 |
| - Adapted/translated information for (e.g.) non-English speakers or those with learning disabilities | 1 - Highly effective (13/37) | N/A (12/31) | N/A (11/33) | 0.478 | 0.147 (0.019) | Code 6 (33/101) | Beta=0.116, OR=1.12, p=0.201 | Beta=0.084, OR=1.09, p=0.373 | Beta=0.188, OR=1.21, p=0.046 |
| In your experience, do local specialist diabetes teams engage in outreach to support people experiencing homelessness with diabetes? | No (21/39) |  | No (16/33) | 0.894 | 0.960 (-0.014) | No (37/72) | Beta=0.176, OR=1.19, p=0.589 | Beta=-0.230, OR=0.79, p=0.475 | Beta=0.483, OR=1.62, p=0.134 |
| Does your service engage in outreach efforts to support people experiencing homelessness who have diabetes? |  | No (13/32) |  |  |  |  | Beta=0.108, OR=1.11, p=0.797 | Beta=0.801, OR=2.23, p=0.058 | Beta=0.170, OR=1.18, p=0.703 |
| Are you aware of any specialist homeless/Inclusion health services in your local area? |  | Yes (22/32) |  |  |  |  | Beta=0.188, OR=1.21, p=0.592 | Beta=0.470, OR=1.60, p=0.173 | Beta=0.507, OR=1.66, p=0.216 |
| Please indicate how often you contact these services with regards to providing diabetes care/support for people who are experiencing homelessness. |  | Sometimes (7/22) |  |  |  |  | Beta=0.507, OR=1.66, p=0.122 | Beta=0.893, OR=2.44, p=0.011 | Beta=0.373, OR=1.45, p=0.264 |
| Have you received specialised training on providing diabetes care and/or support for people with complex needs (e.g. experiencing homelessness, substance misuse needs, mental health needs, safeguarding concerns). | No (35/39) | No (31/32) | No (29/33) | 0.393 | 0.397 (-0.002) | No (95/104) | Beta=1.069, OR=2.91, p=0.091 | Beta=0.956, OR=2.60, p=0.138 | Beta=0.926, OR=2.52, p=0.130 |
| Please indicate how recently you received this training: | Within the past 6 months (2/4) | 2 to 5 years ago (1/1) | 1 to 2 years ago (2/4) | 0.383 | 0.193 (0.215) | Within the past 6 months (3/9) | Beta=0.954, OR=2.60, p=0.040 | Beta=0.481, OR=1.62, p=0.305 | Beta=0.107, OR=1.11, p=0.823 |
| How would you rate the quality of the training you received on a scale of 1 to 5, with 1 being excellent and 5 poor being? | 3 (4/7) | 3 (1/1) | 3 (3/6) | 0.497 | 0.149 (0.164) | 3 (8/14) | Beta=0.465, OR=1.59, p=0.330 | Beta=-0.290, OR=0.75, p=0.504 | Beta=0.509, OR=1.66, p=0.361 |
| Are there clear policies in place at your organisation regarding providing diabetes care and/or support for people experiencing homelessness or those with other complex vulnerabilities? | No (19/38) | No (13/32) | No (17/33) | 0.234 | 0.884 (-0.018) | No (49/103) | Beta=0.484, OR=1.62, p=0.025 | Beta=0.143, OR=1.15, p=0.511 | Beta=0.344, OR=1.41, p=0.123 |
| **On a scale of 1 to 10, where 1 indicates 'Not at all prepared' and 10 indicates 'Extremely prepared,' how would you rate your level of preparedness to provide diabetes care and support for individuals experiencing homelessness, particularly those with complex needs such as mental health issues, safeguarding concerns, or substance misuse needs?** | 5 (8/35) | 5 (8/27) | 6 (9/25) | 0.419 | 0.034 (0.057) | 6 (22/87) | Beta=0.229, OR=1.26, p=0.012 | Beta=0.177, OR=1.19, p=0.058 | **Preparedness to provide care** |
| For each of the following training topics, please select the response that best applies to you. | | | | | | | | | |
| - Nutrition screening and counselling | I would like to do it (25/38) | I would like to do it (15/32) | I would like to do it (18/32) | 0.276 | 0.229 (0.010) | I would like to do it (58/102) | Beta=-0.180, OR=0.83, p=0.304 | Beta=-0.148, OR=0.86, p=0.406 | Beta=-0.064, OR=0.94, p=0.748 |
| - The 9 diabetes processes of care | I would like to do it (24/39) | Completed (23/32) | I would like to do it (22/32) | 0.000 (0.411) | 0.000 (0.282) | I would like to do it (53/103) | Beta=0.016, OR=1.02, p=0.923 | Beta=-0.274, OR=0.76, p=0.114 | Beta=0.222, OR=1.25, p=0.225 |
| - Recognising the difference between type 1, type 2 and type 3 diabetes | I would like to do it (18/39) | Completed (24/32) | I would like to do it (18/32) | 0.003 (0.312) | 0.000 (0.161) | Completed (49/103) | Beta=-0.121, OR=0.89, p=0.470 | Beta=-0.223, OR=0.80, p=0.191 | Beta=0.222, OR=1.25, p=0.211 |
| - Interaction between diabetes and drugs/alcohol | I would like to do it (27/39) | Completed (15/32) | I would like to do it (27/31) | 0.013 (0.281) | 0.001 (0.113) | I would like to do it (69/102) | Beta=0.001, OR=1.00, p=0.997 | Beta=-0.110, OR=0.90, p=0.566 | Beta=0.357, OR=1.43, p=0.082 |
| - Cultural competence in care for homeless populations | I would like to do it (25/39) | I would like to do it (27/32) | I would like to do it (26/32) | 0.314 | 0.223 (0.010) | I would like to do it (78/103) | Beta=-0.063, OR=0.94, p=0.773 | Beta=-0.274, OR=0.76, p=0.215 | Beta=0.072, OR=1.07, p=0.781 |
| - Mental health considerations in diabetes care for homeless individuals | I would like to do it (31/39) | I would like to do it (26/32) | I would like to do it (28/32) | 0.390 | 0.127 (0.021) | I would like to do it (85/103) | Beta=-0.149, OR=0.86, p=0.595 | Beta=-0.461, OR=0.63, p=0.111 | Beta=0.206, OR=1.23, p=0.515 |
| - Outreach and access to care for homeless individuals with diabetes | I would like to do it (26/39) | I would like to do it (24/31) | I would like to do it (24/31) | 0.595 | 0.445 (-0.004) | I would like to do it (74/101) | Beta=0.309, OR=1.36, p=0.166 | Beta=0.172, OR=1.19, p=0.441 | Beta=0.859, OR=2.36, p=0.001 |
| - Smoking cessation and diabetes care | I would like to do it (20/39) | Completed (19/32) | I would like to do it (14/30) | 0.007 (0.296) | 0.001 (0.126) | I would like to do it (44/101) | Beta=-0.100, OR=0.91, p=0.523 | Beta=-0.042, OR=0.96, p=0.794 | Beta=0.192, OR=1.21, p=0.267 |

* Beta Coefficient (β): The β coefficient represents the change in the outcome variable for a one-unit change in the predictor variable, assuming that all other variables in the model are held constant. In regression models, a positive β indicates that as the predictor increases, the outcome variable also increases. Conversely, a negative β suggests that as the predictor increases, the outcome variable decreases. The magnitude of the β coefficient indicates the strength of the effect. A larger absolute value of β suggests a stronger effect.

** Odds Ratio (OR): The Odds Ratio is a measure of association between an exposure and an outcome. It represents the odds that the outcome will occur given a particular exposure, compared to the odds of the outcome occurring without the exposure. An OR of 1 indicates no difference in odds between the two groups. An OR greater than 1 indicates that the exposure is associated with higher odds of the outcome. An OR less than 1 suggests that the exposure is associated with lower odds of the outcome. The further the OR is from 1, whether above or below, the stronger the association between the exposure and the outcome.
