# Supplementary material for: Diabetes care for people experiencing homelessness in the UK: insights from a national survey of frontline professionals and the development of an integrated care model
Source: Front Public Health. 2025 Oct 13;13:1672014. doi: 10.3389/fpubh.2025.1672014 (PMC12554705; doi:10.3389/fpubh.2025.1672014)
Supplement: Supplementary file 3 [file Data_Sheet_1.pdf]

Group specific	Question	Text			Option1	Option2	Option3	Option4	Option5	Option6	Option7	Option8	Option9	Option10	Option11	Option12	Option13	Option14	Option15
	Q1	**I agree to all of the above.**	General Agreement	Comment	Yes														
Total	Q2	**Please select the most appropriate description of your work with people experiencing homelessness:**	Work with Homeless	Grouping Variable	I work in a Specialist Homeless Health/ Inclusion Health service.	I work in a specialist diabetes service.	I work in another role through which I have come into contact with people experiencing homelessness who have diabetes.												
HIS, SDS	Q3	**Please indicate which type of setting you primarily work in (You may tick more than one if applicable)**	Work Setting	Demographics	Acute Hospital Trust	Mental Health Trust	Clinics/Outpatient Services	Community Health Center	Community Team	Mobile health unit	Primary Care/GP	Other							
HIS, SDS	Q4	**Please specify:**	Details																
HIS	Q5	**Please indicate the type of service you work for (You may tick more than one if applicable)**	Service Type	Demographics	Homeless health peer advocacy service	Specialist addictions services	Specialist Community Health Visiting services for families	Specialist Community nursing services for single adults	Specialist GP practice	Specialist hospital services	Specialist mental health services	Specialist step-down services	Street outreach bus or other services	Don't know	Other				
HIS	Q6	**Please specify:**		Demographics															
HCP	Q7	**Please indicate the type of organisation you work for.**	Organisation Type	Demographics	NHS Healthcare Organisation	Non-NHS Healthcare Organisation	Third Sector Organisation	Local Authority Service	Other										
HCP	Q8	**Please describe the type of organisatition you work for:**	Organisation Description	Demographics															
HCP	Q9	**Please describe your job role:**	Job Role	Demographics															
Total	Q10	**Please indicate your professional background (You may tick more than one if applicable)**	Professional Background	Demographics	Counsellor / Psychotherapist	Dentist	Dietitian	Health Care Assistant	Medical Doctor / General Practitioner	Mental health practitioner	Nurse	Occupational therapist	Optician/ Optometrist	Physiotherapist	Podiatrist/Chiropodist	Psychiatrist	Psychologist	Social Worker	Other
Total	Q11	**Please specify:**		Demographics															
Total	Q12	**How many years of experience do you have in your profession?**	Years of Experience	Demographics															
Total	Q13	**How many years of experience do you have working with people experiencing homelessness?**	Homelessness Experience	Demographics															
Total	Q14	**How many years of experience do you have working in your current role?**	Current Role Experience	Demographics															
Total	Q15	**Please select the region where you primarily work:**	Work Region	Demographics	North East England	North West England	Yorkshire and the Humber	East Midlands	West Midlands	East of England	London	South East England	South West England						
Total	Q16	**Please indicate if your service is located in a rural or urban area.**	Location Type	Demographics	Rural	Urban													
Total	Q17	**Please share any additional comments about your professional background, if you feel necessary:**	Additional Comments	Demographics															
HIS, HCP	Q18	**In the past 12 months, how often have you worked with people experiencing homelessness who have diabetes at your service?**	Diabetes Care Frequency	Prevalence	All the time	Often	Sometimes	Rarely	Never	Don't Know/Unsure									
SDS	Q19	**In the past 12 months, how often have you worked with people experiencing homelessness at your service?**	Homeless Service Frequency	Prevalence	All the time	Often	Sometimes	Rarely	Never	Don't Know/Unsure									
Total	Q20	**In the past 3 months, how many people experiencing homelessness with diabetes have you worked with?**	Diabetic Homeless Count	Prevalence	None	1 to 5	6 to 10	11 to 15	16 to 20	20 +	Don't Know/Unsure								
HIS, HCP	Q21	**In your work with individuals experiencing homelessness who have diabetes, how many do you estimate require support from a specialist diabetes team?**	Specialist Support Estimate	Prevalence	All	Most	Some	A few	None	I don't have this information.									
HIS, HCP	Q22	**Of those patients requring support from a specialist diabetes team, how many do you think received a referral to such a team?**	Specialist Referral Estimate	Prevalence	All	Most	Some	A Few	None	I don't have this information.									
HIS, HCP	Q23	**Of these patients requiring a referral but not receiving one, please describe why you think this is the case, if you feel able to do so.**	Unreferred Reason	Prevalence															
SDS	Q24	**Of those who were referred to specialist diabetes teams, how many do you think accessed the service and received care?**	Care Access After Referral	Prevalence	All	Most	Some	Few	None	I don't have this information.									
HIS, HCP	Q25	**Of these patients receiving a referral, but not accessing and receiving care, please describe why you think this is the case, if you feel able to do so.**	Non-Access Reason	Prevalence															
Total	Q26	**Type 1 (%)**	Type 1 Diabetes Proportion	Prevalence															
Total	Q27	**Type 2/ 3 (%)**	Type 2/3 Diabetes Proportion	Prevalence															
Total	Q28	**During the past 12 months, have you worked with people experiencing homelessness who required insulin therapy?**	Insulin Therapy Encounter	Prevalence	Yes	No	Don't Know/ Unsure												
Total	Q29	**Please share any additional comments on this section below:**	Additional Section Comments	Prevalence															
Total	Q30	**Based on your professional experience, how would you rate diabetes care outcomes for individuals experiencing homelessness who have diabetes?**	Diabetes Care Rating	Care outcome	Excellent - Consistently successful management and improvement in patients' condition	Good - Generally effective management with occasional setbacks	Average - Adequate management, but with room for improvement	Poor - Inconsistent management, leading to frequent complications or deterioration	Very Poor - Inadequate management, resulting in severe complications or worsening of the patients' condition	I don't have this information.									
Total	Q31	**Please share any additional comments:**	Additional Comments	Care outcome															

Total	Q32	**Based on your professional experience, how frequently do you encounter diabetes-related complications amomgst those experiencing homelessness with diabetes, compared to those in the general population with diabetes?**	Complication Frequency Comparison	Diabetes Related Compications	Significantly more often	Slightly more often	About the same	Slightly less often	Significantly less often	I don't have this information.									
Total	Q33	**Based on your professional experience, how frequently do you encounter the following issues amongst patients who are experiencing homelessness with diabetes? (Please rate on a scale from 'Very often' to 'Never', using the provided matrix)**	Encountered Issues	Diabetes Related Compications															
Total	Q33_1	**Leg or foot amputations**	Amputation Frequency	Diabetes Related Compications	Very frequently	Frequently	Occasionally	Rarely	Never	I don't have this information.									
Total	Q33_2	**Vision problems or loss**	Vision Problem Frequency	Diabetes Related Compications	Very frequently	Frequently	Occasionally	Rarely	Never	I don't have this information.									
Total	Q33_3	**Cardiovascular issues (e.g., heart disease, stroke)**	Cardiovascular Issue Frequency	Diabetes Related Compications	Very frequently	Frequently	Occasionally	Rarely	Never	I don't have this information.									
Total	Q33_4	**Kidney disease or damage**	Kidney Disease Frequency	Diabetes Related Compications	Very frequently	Frequently	Occasionally	Rarely	Never	I don't have this information.									
Total	Q33_5	**Dental issues (e.g., abcesses)**	Dental Issue Frequency	Diabetes Related Compications	Very frequently	Frequently	Occasionally	Rarely	Never	I don't have this information.									
Total	Q33_6	**Sexual problems (e.g., impotence)**	Sexual Problem Frequency	Diabetes Related Compications	Very frequently	Frequently	Occasionally	Rarely	Never	I don't have this information.									
Total	Q33_7	**Other complications (please specify in the provided free text field)**	Other Complications	Diabetes Related Compications	Very frequently	Frequently	Occasionally	Rarely	Never	I don't have this information.									
Total	Q34	**Please describe any other diabetes-related complications you have encountered amongst patients experiencing homelessness.**	Other Diabetes Complications	Diabetes Related Compications															
Total	Q35	**Please share any additional comments on this section below:**	Additional Complications Comments	Diabetes Related Compications															
HIS, HCP	Q36	**Do your patients experiencing homelessness get screened for diabetes as part of your standard assessment process?**	Diabetes Screening Standard	Standard of practice	Yes	No	Don't Know/ Unsure												
HIS, HCP	Q37	**Please briefly describe how this is done:**	Screening Process Description	Standard of practice															
SDS	Q38	**Do you assess the housing status of new referrals, as part of your standard assessment process?**	Housing Status Assessment	Standard of practice	Yes	No	Unsure	I don't have this information											
SDS	Q39	**Do you receive information regarding the housing status of newly referred patients, as part of your standard referral process?**	Referral Housing Status	Standard of practice	Yes	No	Unsure												
Total	Q40	**For each of the following types of diabetes screening, please indicate how easy it is for patients experiencing homelessness with diabetes to access them within the diabetes care process. (Please rate on a scale from "Very Easy" to "Very Difficult" for each screening type, matrix)**	Diabetes Screening Accessibility	Access to service															
Total	Q40_1	**Hemoglobin A1c (HbA1c) Test:**	HbA1c Test Accessibility	Access to service	Very Easy	Somewhat Easy	Neutral	Somewhat Difficult	Very Difficult	I don't have this information.									
Total	Q40_2	**Fasting Blood Sugar (FBS) Test**	FBS Test Accessibility	Access to service	Very Easy	Somewhat Easy	Neutral	Somewhat Difficult	Very Difficult	I don't have this information.									
Total	Q40_3	**Oral Glucose Tolerance Test (OGTT)**	OGTT Accessibility	Access to service	Very Easy	Somewhat Easy	Neutral	Somewhat Difficult	Very Difficult	I don't have this information.									
Total	Q40_4	**Random Blood Sugar Test**	Random Blood Sugar Test Accessibility	Access to service	Very Easy	Somewhat Easy	Neutral	Somewhat Difficult	Very Difficult	I don't have this information.									
Total	Q40_5	**Blood Pressure Check**	Blood Pressure Check Accessibility	Access to service	Very Easy	Somewhat Easy	Neutral	Somewhat Difficult	Very Difficult	I don't have this information.									
Total	Q40_6	**Foot Examination**	Foot Examination Accessibility	Access to service	Very Easy	Somewhat Easy	Neutral	Somewhat Difficult	Very Difficult	I don't have this information.									
Total	Q40_7	**Eye Examination**	Eye Examination Accessibility	Access to service	Very Easy	Somewhat Easy	Neutral	Somewhat Difficult	Very Difficult	I don't have this information.									
Total	Q40_8	**Kidney Function Test**	Kidney Function Test Accessibility	Access to service	Very Easy	Somewhat Easy	Neutral	Somewhat Difficult	Very Difficult	I don't have this information.									
Total	Q40_9	**Nutrition screening using a recommended tool**	Nutrition Screening Accessibility	Access to service	Very Easy	Somewhat Easy	Neutral	Somewhat Difficult	Very Difficult	I don't have this information.									
Total	Q41	**Please describe any barriers you have experienced, with regards to securing/providing different diabetes screenings for people who are experiencing homelessness.**	Diabetes Screening Barriers	Access to service															
Total	Q42	**For patients experiencing homelessness with diabetes, please indicate how easy it was for them to access the following types of health support (for those it is relevant to, for example cessation for smokers, alcohol team referrals for those with alcohol issues).**	Health Support Accessibility	Access to service															
Total	Q42_1	**Smoking Cessation Support**	Smoking Cessation Support Accessibility	Access to service	Very Easy	Somewhat Easy	Neither Easy nor Difficult	Somewhat Difficult	Very Difficult	I don't have this information.									
Total	Q42_2	**Alcohol & Drug Misuse Support**	Alcohol & Drug Support Accessibility	Access to service	Very Easy	Somewhat Easy	Neither Easy nor Difficult	Somewhat Difficult	Very Difficult	I don't have this information.									
Total	Q42_3	**Dietician Support**	Dietician Support Accessibility	Access to service	Very Easy	Somewhat Easy	Neither Easy nor Difficult	Somewhat Difficult	Very Difficult	I don't have this information.									
Total	Q42_4	**Mental Health Support**	Mental Health Support Accessibility	Access to service	Very Easy	Somewhat Easy	Neither Easy nor Difficult	Somewhat Difficult	Very Difficult	I don't have this information.									

Total	Q42_5	**Exercise on Prescription**	Exercise Prescription Accessibility	Access to service	Very Easy	Somewhat Easy	Neither Easy nor Difficult	Somewhat Difficult	Very Difficult	I don't have this information.									
Total	Q43	**Please share any additional comments on this section below:**	Additional Health Support Comments	Access to service															
Total	Q44	**Based on your professional experience, how difficult have you found managing diabetes for your patients who are experiencing homelessness / getting diabetes managed for your clients if you are a support worker?**	Diabetes Management Difficulty	Care outcome	Very challenging	Somewhat challenging	Neither challenging nor easy	Somewhat easy	Easy	Unsure	I don't have this information.								
Total	Q45	**In your experience, how have the following barriers hindered patients experiencing homelessness from accessing diabetes care?**	Care Access Barriers																
Total	Q45_1	**Alcohol/drug misuse/other complex needs**	Alcohol/Drug Misuse Barrier		Never a barrier	Rarely a barrier	Sometimes a barrier	Often a barrier	Always a barrier										
Total	Q45_2	**Patient fear or mistrust of healthcare providers**	Patient Fear Barrier		Never a barrier	Rarely a barrier	Sometimes a barrier	Often a barrier	Always a barrier										
Total	Q45_3	**Patient lack of understanding of diabetes**	Patient Understanding Barrier		Never a barrier	Rarely a barrier	Sometimes a barrier	Often a barrier	Always a barrier										
Total	Q45_4	**Patient lack of awareness of diabetes**	Patient Awareness Barrier		Never a barrier	Rarely a barrier	Sometimes a barrier	Often a barrier	Always a barrier										
Total	Q45_5	**Inflexible appointment times**	Appointment Time Flexibility		Never a barrier	Rarely a barrier	Sometimes a barrier	Often a barrier	Always a barrier										
Total	Q45_6	**Financial constraints (e.g. being unable to pay for transport)**	Financial Constraint Barrier		Never a barrier	Rarely a barrier	Sometimes a barrier	Often a barrier	Always a barrier										
Total	Q45_7	**Other**			Never a barrier	Rarely a barrier	Sometimes a barrier	Often a barrier	Always a barrier										
Total	Q46	**Please describe any other barriers that people experiencing homelessness face when engaging with their diabetes care.**	Other Care Barriers																
Total	Q47	**In your experience, how often have the following factors acted as barriers to providing quality diabetes care for patients experiencing homelessness?**	Diabetes Care Barriers																
Total	Q47_1	**Insufficient training around providing diabetes care/support for people experiencing homelessness**	Training Insufficiency Barrier		Never a barrier	Rarely a barrier	Sometimes a barrier	Often a barrier	Always a barrier	I'm not sure									
Total	Q47_2	**Limited resources (e.g., funding, supplies, etc.)**	Resource Limitation Barrier		Never a barrier	Rarely a barrier	Sometimes a barrier	Often a barrier	Always a barrier	I'm not sure									
Total	Q47_3	**Complex needs of patients (e.g. substance misuse, mental health)**	Patient Complex Needs Barrier		Never a barrier	Rarely a barrier	Sometimes a barrier	Often a barrier	Always a barrier	I'm not sure									
Total	Q47_4	**Difficulty contacting patients for check-ups/follow-ups**	Patient Contact Difficulty		Never a barrier	Rarely a barrier	Sometimes a barrier	Often a barrier	Always a barrier	I'm not sure									
Total	Q47_5	**Difficulty securing/providing diabetes screenings for patients**	Screening Provision Difficulty		Never a barrier	Rarely a barrier	Sometimes a barrier	Often a barrier	Always a barrier	I'm not sure									
Total	Q47_6	**Difficulty working collaboratively with other relevant services**			Never a barrier	Rarely a barrier	Sometimes a barrier	Often a barrier	Always a barrier	I'm not sure									
Total	Q47_7	**Other**			Never a barrier	Rarely a barrier	Sometimes a barrier	Often a barrier	Always a barrier	I'm not sure									
Total	Q48	**Please describe any other barriers that practitioners face when providing diabetes care/support for people experiencing homelessness.**	Collaboration Difficulty																
Total	Q49	**Please rate the extent to which the following improvements/ support would help you to overcome challenges in providing diabetes care to people experiencing homelessness (1 Very helpful - 5 Not helpful at all)**	Improvement Rating																
Total	Q49_1	**Additional resources for services (funding, staff, equipment etc.)**	Resources for Services		1 - Very helpful	2	3	4	5 - Not helpful at all	I'm not sure									
Total	Q49_2	**Training/education for healthcare staff on providing diabetes care for PEH**	Staff Training		1 - Very helpful	2	3	4	5 - Not helpful at all	I'm not sure									
Total	Q49_3	**Improved accessibility for healthcare appointments (e.g. flexible times)**	Appointment Accessibility		1 - Very helpful	2	3	4	5 - Not helpful at all	I'm not sure									
Total	Q49_4	**Enhanced collaboration between healthcare and community organisations**	Healthcare-Community Collaboration		1 - Very helpful	2	3	4	5 - Not helpful at all	I'm not sure									
Total	Q49_5	**Enhanced collaboration between specialist and mainstream healthcare organisations**	Healthcare-Organisations Collaboration		1 - Very helpful	2	3	4	5 - Not helpful at all	I'm not sure									
Total	Q49_6	**Greater availability of educational materials on diabetes for patients**	Educational Materials		1 - Very helpful	2	3	4	5 - Not helpful at all	I'm not sure									
Total	Q49_7	**Improved data sharing between relevant organisations**	Data Sharing		1 - Very helpful	2	3	4	5 - Not helpful at all	I'm not sure									
Total	Q49_8	**Other**	Other Improvements		1 - Very helpful	2	3	4	5 - Not helpful at all	I'm not sure									
Total	Q50	**If you selected 'Other', please describe this resource in the box provided.**	Other Resources																
Total	Q51	**Please describe any service improvements/examples of best practise that you have encountered with regards to improving diabetes care outcomes for patients experiencing homelessness.**	Best Practice Examples																
Total	Q52	**Please share any additional comments on this section below:**	Additional Improvements Comments																

Total	Q53	**Which strategies do you currently use to engage patients experiencing homelessness in their diabetes care? Please select all that apply and rate the effectiveness of each strategy using the scale provided**	Engagement Strategies																
Total	Q53_1	**Outreach programs**	Outreach Program Effectiveness		1 - Not effective	2	3	4	5 - Highly effective	N/A									
Total	Q53_2	**Mobile health services**	Mobile Health Services Effectiveness		1 - Not effective	2	3	4	5 - Highly effective	N/A									
Total	Q53_3	**Peer support and mentoring**	Peer Support Effectiveness		1 - Not effective	2	3	4	5 - Highly effective	N/A									
Total	Q53_4	**Health promotion and education**	Education Effectiveness		1 - Not effective	2	3	4	5 - Highly effective	N/A									
Total	Q53_5	**Multi-disciplinary case conferences and care coordination**	Case Conferences Effectiveness		1 - Not effective	2	3	4	5 - Highly effective	N/A									
Total	Q53_6	**Telemedicine or remote monitoring**	Telemedicine Effectiveness		1 - Not effective	2	3	4	5 - Highly effective	N/A									
Total	Q53_7	**Flexible appointment times**	Flexible Times Effectiveness		1 - Not effective	2	3	4	5 - Highly effective	N/A									
Total	Q53_8	**Adapted/translated information for (e.g.) non-English speakers or those with learning disabilities**	Adapted Information Effectiveness		1 - Not effective	2	3	4	5 - Highly effective	N/A									
Total	Q54	**Please describe any other strategies you use to improve accessibility and engagement for people experiencing homelessness with diabetes and/or any other strategies you feel are effective at improving service accessibility.**	Other Engagement Strategies																
HIS, HCP	Q55	**In your experience, do local specialist diabetes teams engage in outreach to support people experiencing homelessness with diabetes?**	Specialist Outreach		Yes	No	I don't have this information												
SDS	Q56	**Does your service engage in outreach efforts to support people experiencing homelessness who have diabetes?**	Service Outreach		Yes	No	I don't have this information												
SDS	Q57	**Are you aware of any specialist homeless/Inclusion health services in your local area?**	Specialist Services Awareness		Yes	No	I don't have this information												
SDS	Q58	**Please indicate how often you contact these services with regards to providing diabetes care/support for people who are experiencing homelessness.**	Specialist Contact Frequency		All the time	Often	Sometimes	Rarely	Never	I don't know									
Total	Q59	**Please share any additional comments or insights regarding your experience in providing diabetes care for homeless individuals.**	Additional Outreach Comments																
Total	Q60	**Have you received specialised training on providing diabetes care and/or support for people with complex needs (e.g. experiencing homelessness, substance misuse needs, mental health needs, safeguarding concerns).**	Specialised Training		Yes	No	I am not sure												
Total	Q61	**Please indicate how recently you received this training:**	Training Recency		Within the past 6 months	6 to 12 months ago	1 to 2 years ago	2 to 5 years ago	Over 5 years ago										
Total	Q62	**How would you rate the quality of the training you received on a scale of 1 to 5, with 1 being poor and 5 being excellent?**	Training Quality Rating																
Total	Q63	**Are there clear policies in place at your organisation regarding providing diabetes care and/or support for people experiencing homelessness or those with other complex vulnerabilities?**	Policies Clarity		Yes	No	Unsure	I don't have that information.											
Total	Q64	**On a scale of 1 to 10, where 1 indicates 'Not at all prepared' and 10 indicates 'Extremely prepared,' how would you rate your level of preparedness to provide diabetes care and support for individuals experiencing homelessness, particularly those with complex needs such as mental health issues, safeguarding concerns, or substance misuse needs?**	Preparedness Rating																
Total	Q65	**For each of thee following training topics, please select the response that best applies to you.**	Training Topic Preference																
Total	Q65_1	**Nutrition screening and counseling**	Nutrition Screening Training		I have completed training on this topic	I am currently undergoing training on this topic	I haven't received training on this topic but I would like to	I haven't received training on this topic and wouldn't like to											
Total	Q65_2	**The 9 diabetes processes of care**	Diabetes Care Processes Training		I have completed training on this topic	I am currently undergoing training on this topic	I haven't received training on this topic but I would like to	I haven't received training on this topic and wouldn't like to											
Total	Q65_3	**Recognising the difference between type 1, type 2 and type 3 diabetes**	Diabetes Types Training		I have completed training on this topic	I am currently undergoing training on this topic	I haven't received training on this topic but I would like to	I haven't received training on this topic and wouldn't like to											

Total	Q65_4	**Interaction between diabetes and drugs/alcohol**	Diabetes and Drugs/Alcohol Interaction Training		I have completed training on this topic	I am currently undergoing training on this topic	I haven't received training on this topic but I would like to	I haven't received training on this topic and wouldn't like to											
Total	Q65_5	**Cultural competence in care for homeless populations**	Cultural Competence Training		I have completed training on this topic	I am currently undergoing training on this topic	I haven't received training on this topic but I would like to	I haven't received training on this topic and wouldn't like to											
Total	Q65_6	**Mental health considerations in diabetes care for homeless individuals**	Mental Health Considerations Training		I have completed training on this topic	I am currently undergoing training on this topic	I haven't received training on this topic but I would like to	I haven't received training on this topic and wouldn't like to											
Total	Q65_7	**Outreach and access to care for homeless individuals with diabetes**	Outreach and Access Training		I have completed training on this topic	I am currently undergoing training on this topic	I haven't received training on this topic but I would like to	I haven't received training on this topic and wouldn't like to											
Total	Q65_8	**Smoking cessation and diabetes care**	Smoking Cessation Training		I have completed training on this topic	I am currently undergoing training on this topic	I haven't received training on this topic but I would like to	I haven't received training on this topic and wouldn't like to											
Total	Q65_9	**Other**	Other Training		I have completed training on this topic	I am currently undergoing training on this topic	I haven't received training on this topic but I would like to	I haven't received training on this topic and wouldn't like to											
Total	Q66	**Please specify any other topics you would like to receive training on.**																	
Total	Q67	**Please select the format of training you would prefer for learning about diabetes care and/or support for people experiencing homelessness. (Select all that apply)**	Preferred Training Format		In-person workshop or seminar	Online course or webinar	Self-paced learning modules	Online discussion forums	Interactive case studies or simulations	Other									
Total	Q68	**Please describe any other training topics you think are relevant for improving diabetes care and outcomes for people experiencing homelessness.**	Relevant Training Topics																
Total	Q69	**Please share any additional thoughts or suggestions on how to improve diabetes care for homeless individuals, in relation to your training and education.**	Additional Training Comments																
